# Exploring the genomic traits of fungus-feeding bacterial genus *Collimonas*

**DOI:** 10.1186/s12864-015-2289-3

**Published:** 2015-12-24

**Authors:** Chunxu Song, Ruth Schmidt, Victor de Jager, Dorota Krzyzanowska, Esmer Jongedijk, Katarina Cankar, Jules Beekwilder, Anouk van Veen, Wietse de Boer, Johannes A. van Veen, Paolina Garbeva

**Affiliations:** Netherlands Institute of Ecology, Department of Microbial Ecology, Droevendaalsesteeg 10, Wageningen, 6708 PB The Netherlands; Laboratory of Biological Plant Protection, Intercollegiate Faculty of Biotechnology UG&MUG, Kladki 24, Gdansk, 80-822 Poland; Laboratory of Plant Physiology, Wageningen University, Droevendaalsesteeg 1, Wageningen, 6708 PB The Netherlands; Business Unit Bioscience, Plant Research International, Wageningen University and Research Centre, Wageningen, The Netherlands

**Keywords:** Comparative genomics, *Collimonas*, Secondary metabolites, Terpenes

## Abstract

**Background:**

*Collimonas* is a genus belonging to the class of Betaproteobacteria and consists mostly of soil bacteria with the ability to exploit living fungi as food source (mycophagy). *Collimonas* strains differ in a range of activities, including swimming motility, quorum sensing, extracellular protease activity, siderophore production, and antimicrobial activities.

**Results:**

In order to reveal ecological traits possibly related to *Collimonas* lifestyle and secondary metabolites production, we performed a comparative genomics analysis based on whole-genome sequencing of six strains representing 3 recognized species. The analysis revealed that the core genome represents 43.1 to 52.7 % of the genomes of the six individual strains. These include genes coding for extracellular enzymes (chitinase, peptidase, phospholipase), iron acquisition and type II secretion systems. In the variable genome, differences were found in genes coding for secondary metabolites (e.g. tripropeptin A and volatile terpenes), several unknown orphan polyketide synthase-nonribosomal peptide synthetase (PKS-NRPS), nonribosomal peptide synthetase (NRPS) gene clusters, a new lipopeptide and type III and type VI secretion systems. Potential roles of the latter genes in the interaction with other organisms were investigated. Mutation of a gene involved in tripropeptin A biosynthesis strongly reduced the antibacterial activity against *Staphylococcus aureus*, while disruption of a gene involved in the biosynthesis of the new lipopeptide had a large effect on the antifungal/oomycetal activities.

**Conclusions:**

Overall our results indicated that *Collimonas* genomes harbour many genes encoding for novel enzymes and secondary metabolites (including terpenes) important for interactions with other organisms and revealed genomic plasticity, which reflect the behaviour, antimicrobial activity and lifestylesof *Collimonas* spp.

**Electronic supplementary material:**

The online version of this article (doi:10.1186/s12864-015-2289-3) contains supplementary material, which is available to authorized users.

## Background

The genus *Collimonas* comprises soil bacteria with the ability to grow at the expense of living fungal hyphae under nutrient-limited conditions [1–3]. Since the first description of *Collimonas*, more mycophagous bacteria have been detected [4], but *Collimonas* species are still highly interesting in view of the interactions between bacteria and fungi in soil and the associated ecosystem functions including suppression of pathogens and the production of novel bioactive compounds.

*Collimonas* belongs to the family *Oxalobacteraceae*, class Betaproteobacteria. The first *Collimonas* isolates were obtained within the framework of a project searching for a naturally occurring biocontrol agent of fungi pathogenic to marram grass (*Ammophilia arenaria*) and were determined as being dominant among the cultivable chitinolytic bacteria in the acidic Dutch dune soils [3]. Three species have been described so far: *C. fungivorans*, *C. pratensis* and *C. arenae* [5]. All three species display the ability to feed on fungi (mycophagy), to degrade chitin and to dissolve minerals (weathering) [2]. However, *Collimonas* strains differ in important ecological traits such as colony morphology, the ability to oxidize various carbon sources, in their antibacterial, antifungal and antioomycetal activities [6, 7]. A comparative genomic approach would help to reveal the genetic basis of these ecological differences. Applying a comparative genomic hybridization approach [7] showed that a gene cluster involved in the production of an antifungal polyyne was only found in the genome of a few *Collimonas* strains. The study of Mela et al. [7] was biased in the sense that the hybridization assay used allowed only to screen for absence/presence of genes in other strains as compared to *C. fungivorans* strain Ter 331, the only strain for which the complete genome was sequenced at that time. To reveal the real plasticity of *Collimonas* strains more genome sequences are needed. This will demonstrate constant and variable genetic elements, and hence determine the adaptations of *Collimonas* species and traits important for inter-specific microbial interactions in the soil. To date, only two genome sequences of *Collimonas* are publicly available [7, 8]. Here we report on full genome sequences of five *Collimonas* strains across the three recognized species and performed comparative genome analysis including the recently published *C. fungivorans* Ter331 genome [7]. Gene clusters with potential relevance for interactions of the *Collimonas* species with fungi and other microorganisms were further investigated by gene knock-out mutations and/or enzymatic characterization.

## Results and discussion

### Genomic features

A genome sequence analysis was performed for strains Ter6, Ter91, Ter291, Ter10 and Ter282, using a combined strategy of Illumina Hiseq and PacBio sequencing. A summary of the general genomic features (size, GC content, predicted number of coding sequences, and number of rRNAs) of each *Collimonas* strain is presented in Table 1. Considerable variation in genome size and differences in plasmid content was observed. The six genomes vary in size by approximately one megabase (ranging from 4.7–5.7 Mb) with the number of coding sequences (CDSs) ranging from 4436 to 5424, indicating substantial strain-to-strain variation. The genomes of *C. fungivorans* and *C. pratensis* are larger and have higher GC content than the two strains of *C. arenae*. Only strain *C. fungivorans* Ter331 has a plasmid, described in detail by Mela et al. [9]. Despite the absence of a plasmid, *C. pratensis* Ter91 has the largest genome size (5.7 kb) and highest number of encoding genes (5424), which is likely due to the large number of horizontally acquired genes as indicated by the number of genomic islands (see below).

**Table 1:** *Collimonas* strains and their genomic features

Feature	*Collimonas fungivorans*		*Collimonas pratensis*		*Collimonas arenae*	
	Ter331	Ter6	Ter91	Ter291	Ter10	Ter282
Source	Inner coastal dune soil in Terschelling, the Netherlands					
Chromosome size (Mb)	5.2	5.6	5.7	5.6	4.7	4.7
Plasmid size	40 kb	NA	NA	NA	NA	NA
G + C %	59.6 %	59 %	58.8 %	59 %	56.8 %	56.8 %
Protein-encoding sequences (CDSs)	4910	5233	5424	5228	4436	4473
CDSs on plasmid	44	NA	NA	NA	NA	NA
Hypothetical proteins	1091	1209	1292	1214	1101	991
Average CDS length (nt)	927	923	904	917	834	899
Coding (%)	87.8 %	86.2 %	85.6 %	85.8 %	78.4 %	85.4 %
rRNA	9	9	9	9	9	9
tRNA	52	52	52	51	52	53
contigs	1	1	1	1	1	1

*NA* not applicable, refers to strains in which no plasmids are naturally present

### Phylogenetic analysis

**Fig. 1:**
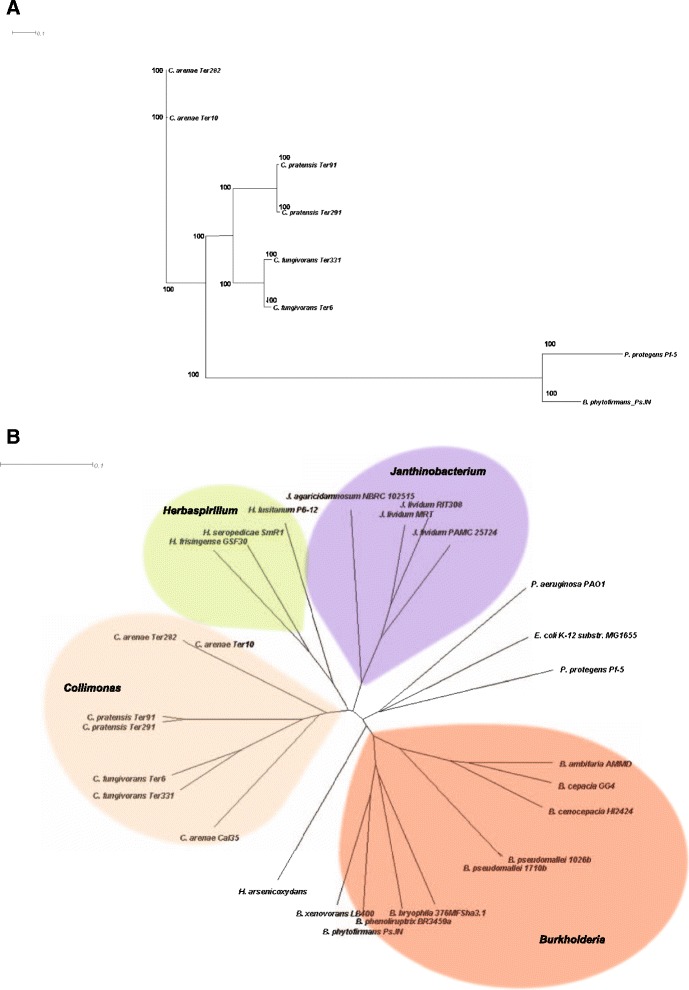
Whole genome phylogeny of the six *Collimonas* genomes. **a** Neighbor-joining tree based on concatenated sequences for 233 protein encoding genes. *Pseudomonas protegens* Pf-5 and *Burkholderia phytofirmans* PsJN were used as outgroup. Bootstrap values are shown on branches. **b** Phylogenetic tree based on a fragmented alignment using BLASTN made with settings 200/100. A dendrogram was produced in SplitsTree 4.13.1 (using neighbor joining method) made from a Nexus file exported from Gegenees. *Burkholderia*, *Janthinobacterium* and *Herbaspirillum* were set as outgroups. Bootstrap values are of all the branches are 100, for clarity reason, not shown in the figure

A phylogenetic tree based on 233 protein-coding genes (Fig. 1a) revealed that *C. fungivorans* and *C. pratensis* are more closely related to each other than to *C. arenae*. Similar clustering was observed based on phylogenetic trees generated with whole genome fragments (200 bp fragment sizes) (Fig. 1b) and 16S rRNA (Additional file 1: Figure S1) where each strain falls into its respective species clade.

### Core and pan-genome analysis

**Fig. 2:**
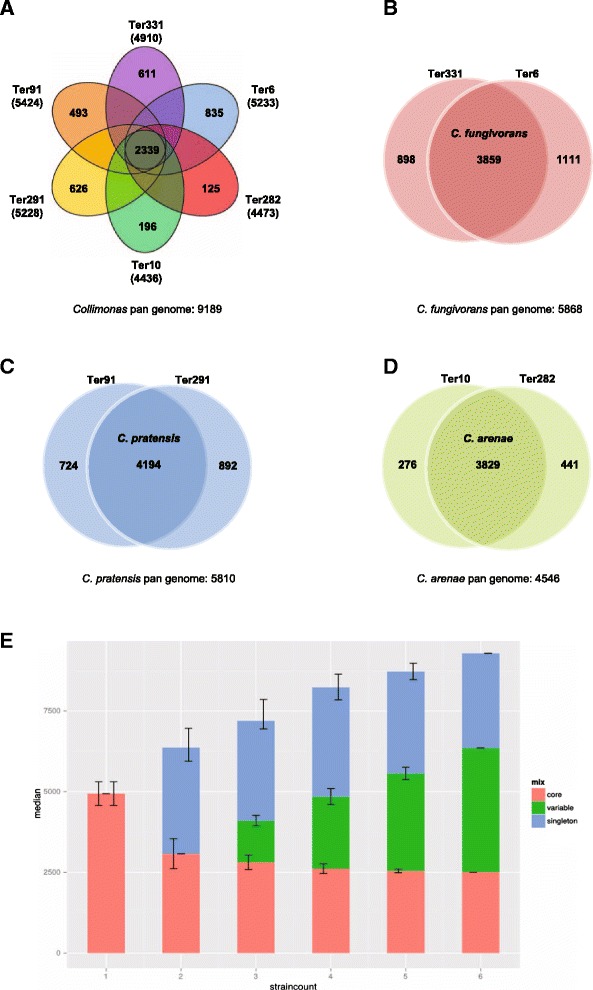
The pan-core genome of *Collimonas* strains. The venn diagrams illustrate the number of shared and unique genes based on clusters of orthologs. **a** Venn diagram showing numbers of species-specific genes commonly found in each genome of each species, (non-overlapping of each oval) and *Collimonas* core orthologous gene number (in the centre). The total number of protein coding genes within each genome is listed below the strain name. **b** Venn diagram showing numbers of unique orthologues genes in *C. fungivorans* strains. **c** Venn diagram showing numbers of unique orthologues genes in *C. pratensis* strains. **d** Venn diagram showing numbers of unique orthologues genes in *C. arenae* strains. **e** Core- and pan-genome as function of the number of genomes taken from the six *Collimonas* genomes in this study. The number of shared and strain specific gene clusters between strains depends on which combinations of strains (x-axis). Specific singleton gene clusters (blue bars) occur only in one strain, variable gene clusters (green bars) occur in more than one but not all strains and core gene clusters (red bars) occur in all strains of a given combination. Error bars represent the standard deviation in the core- (left error bar), variable- (middle error bar) and singleton- (right error bars) gene clusters

**Fig. 3:**
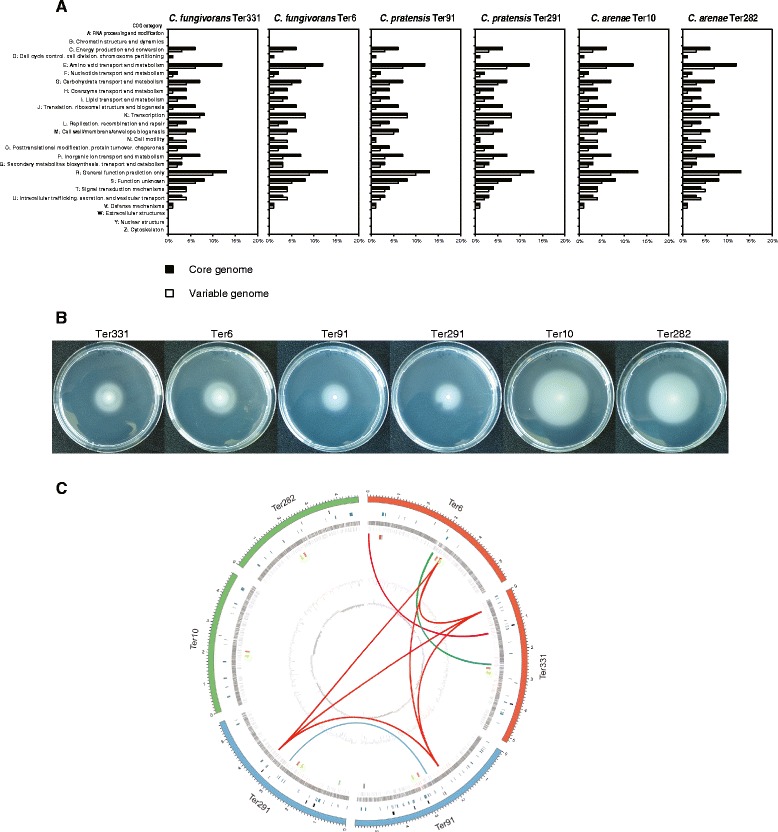
Distribution of orthologous genes based on COG category in each *Collimonas* strain. **a** The percentage of orthologous genes assigned by COG category in the core genome (black bars) and the variable genome (white bars). **b** Swimming motility of *Collimonas* strains Ter331, Ter6, Ter91, Ter291, Ter10 and Ter282 on soft (0.3 % [wt/vol]) agar plates. **c** Comparative genome content of the six *Collimonas* strains. From the outside to the inside circles: Chromosomes of all six strains (red: *C. fungivorans*, blue: *C. pratensis*, green: *C. arenae*). Phages/phage-like regions: black bars. Genomic islands: dark blue bars. Genes in the forward (dark grey) direction, genes in the reverse (light grey) direction, G + C content (dark grey and light grey), GC skew (dark grey: negative values, light grey: positive values). Universal and unique gene clusters are indicated with colored bars (orange: Ornibactin, yellow: Phytoene, red: T3pks-nrps (Ter6), light blue: 2-aa NRPS-1 (Ter6), purple: 2-aa NRPS-2 (Ter291), light green: Nrps-t1pks (HSAF, Ter91)). Shared secondary metabolite gene clusters are indicated with colored lines (deep pink: Collimomycin, dark red: NLP, dark green: Tripropeptin A)

A core genome containing 2339 predicted orthologous groups was identified for the six *Collimonas* strains based on the all-vs-all BLASTp search (Fig. 2a). This core genome represents 43.1 to 52.7 % of the predicted ORF’s of each strain (Fig. 2a), illustrating a large degree of genomic diversity between these strains. Each of the six genomes includes 125 to 835 orthologous groups that are unique (Fig. 2a). Species core orthologous clusters and strain-specific unique clusters within the three *Collimonas* species were examined, respectively (Fig. 2b-d). In the three species, 5868, 5810 and 4546 orthologous clusters were identified and of these, 3859, 4194 and 3829 orthologs were present as the species core genome for *C. fungivorans*, *C. pratensis*, *C. arenae*, respectively (Fig. 2b-d). A core-pan genome evolution plot summarizing the variability in each possible combination of *Collimonas* species shows that the number of unique (singleton) gene clusters is stable. The variable gene clusters are increasing and the core gene clusters are decreasing (Fig. 2e). In order to determine the differences in functions encoded by the core and variable genome of each strain, the proportion of proteins in each COG (Clusters of Orthologous Groups) was plotted versus the COG function. The relative abundance of almost all the COG categories was higher in the core genome of the six strains than in the variable genome. This was not the case for COG categories N (Cell motility), U (Intracellular trafficking, secretion, and vesicular transport) and proteins that cannot be assigned in COG categories (data not shown) where the proteins were more abundant in variable genomes for *C. fungivorans* and *C. arenae* (Fig. 3a). This is most probably due to the fact that a flagellar and chemotaxis-related gene cluster is only present in these two species but not in *C. pratensis* (Additional file 2: Table S1). This finding is consistent with the observed reduced swimming motility of *C. pratensis* Ter91 and Ter291 as compared to the other four strains (Fig. 3b).

Whole genome alignments of the six strains were performed to obtain information on the nucleotide level synteny (Additional file 1: Figure S2). These alignments revealed a very high level of synteny when genomes of strains from the same species were compared (Additional file 1: Figure S2A-C), but many rearrangements and inversions were observed between the genomes of all strains (Additional file 1: Figure S2D).

### Genomic islands (GIs), bacteriophages and CRISPRs

Genomic islands (GIs) are mobile genetic elements acquired by horizontal transfer, which carry multiple genes that are typically involved in pathogenesis or symbiosis. The *Collimonas* genomes carry 7 to 47 GIs ranging from 4.0 kb to 64 kb in size (Fig. 3c). All together, the six *Collimonas* genomes have 139 genomic islands. The large numbers of GIs indicate a complex history of gene recombination and horizontal transfer between bacterial relatives. The genomes of all strains contain one to five possible phages, each ranging in size from 7.0 to 59.9 kb. In total, the six genomes have 18 phages with some of the phages falling into the GIs (Fig. 3c). CRISPRs (Clustered Regularly Interspaced Short Palindromic Repeats) are DNA loci that are involved in prokaryotic immunity to phage infection. Putative CRISPRs were identified using the CRISPRsFinder program [10]. Two confirmed CRISPRs were present only in *C. fungivorans* Ter6 genome. For the other five genomes, no or only questionable CRISPRs were found (data not shown).

### Secretion systems

In Gram-negative bacteria, type II secretion systems (T2SSs) are the most ubiquitous secretion systems used by bacteria to export many extracellular enzymes. T2SSs are conserved and known as a two-step process: proteins are translocated across the inner membrane by the Sec or Tat pathway, and then transported from the periplasm to the exterior by an outer membrane secretin [11]. *SecABDEFY*, *yajC*, *yidC*, *ftsY*, and *ffh* and *tatABC* encoding genes for Sec and Tat pathways respectively were found to be present in all *Collimonas* genomes (Additional file 1: Figure S3A; Additional file 2: Table S2). The outer membrane secretion unit of the T2SS in the *Collimonas* genomes resembles the Gsp system which contains one gene cluster *gspD*-*N*, responsible for secretion of protease, lipase, and phospholipase C in *Burkholderia* [12] (Additional file 1: Figure S3A; Additional file 2: Table S2). A newly described subtype of T2SS, *tad* locus (*t*ight *ad*herence) [13] was identified in all six genomes. The *tad* locus encodes the machinery required for the assembly of adhesive Flp (fimbrial low-molecule-weight protein) pili and is necessary for bacterial adhesion to surfaces, biofilm formation, and pathogenesis as shown for *Aggregatibacter actinomycetemcomitans* [14], *Haemophilus* [15], *Pseudomonas* [16], *Yersinia etc*. [13]. For the mycophagous behavior of *Collimonas*, the adhesion to fungal hyphae might be of prime importance [17] and the presence of the T2SS *tad* locus in all strains indicates that it may be an essential trait for the mycophagy lifestyle of this species.

Type III secretion systems (T3SSs) are used by various Gram-negative bacteria to inject effector proteins into host cells, promoting either mutual benefit or pathogenesis [18, 19] and have been described as important for bacterial interaction with fungi [20]. Three *Collimonas* strains Ter331, Ter6 and Ter91 carry *hrp*-*hrc*1 family gene clusters of T3SS and a second T3SS (Additional file 1: Figure S3A; Additional file 2: Table S2). The T3SSs play crucial role in the virulence of plant and human pathogens [21]. However, their functions in non-pathogenic bacteria are still poorly understood; there are indications that mutation of T3SSs in a plant-growth promoting bacteria *P. fluorescens* SBW25 resulted in a significant reduction in the potential of the bacterium to colonize the root tips of sugar beet seedlings [22]. They might also be involved in bacterial-fungal interactions, facilitating bacteria migration along fungal hyphae [20]. For *Collimonas*, the T3SSs may be important to inject membrane disturbing compounds into the fungal host to get access to nutrients inside the fungal hyphae [2].

Type VI secretion systems (T6SSs) are conserved and prevalent in Gram-negative bacteria. They are known to be involved in competition, predation and inter-specific bacterial interactions [23–25]. In this study, we identified a cluster of genes encoding T6SS only in *C. fungivorans* and *C. praten*sis strains, but not in the *C. arenae* species. This may indicate that horizontal gene transfer events or evolutionary genes loss have occurred. Furthermore, in *C. fungivorans* Ter331, part of the T6SS is located on a genomic island.

Further functional studies are needed to determine the exact role of these secretion systems for *Collimonas* lifestyle and in particular for the attack of fungi.

### Signal transduction systems

Signal transduction systems play important roles for many bacteria enabling them to detect and respond to changes and stresses in the environment [26]. Each *Collimonas* genome encodes 267 to 365 one-component systems (1CSs) which are the majority of signal transduction systems in prokaryotes [27] and 90 to 109 two component system (TCSs) (Additional file 2: Table S3). Additionally, 9 to 29 genes involved in chemotaxis systems were found. Extracytoplasmic function (ECF) sigma factors which comprise the largest group among the σ70 family [27] were also found in all *Collimonas* genomes with numbers ranging from 9 to 11. Higher numbers of signal transduction system were predicted in *C. fungivorans* and *C. pratensis* as compared to *C. arenae*. The overall high number of genes related to signal transduction systems (8–9 % of predicted sequences in the six *Collimonas* genomes) suggest that *Collimonas* possess the ability to sense environmental signals and cues important for their growth, survival and interactions in the heterogeneous and complex soil environment. This is in consistent with the previous report that soil bacteria contain higher number of signal transduction systems as compared to bacteria from a stable environment [28].

Our genomic analysis revealed that all six *Collimonas* genomes contain two QS genes: one autoinducer gene and one luxR-type transcriptional regulator. They show 40 % homology to the CepIR system from *Burkholderia* (Additional file 2: Table S4) which is known to regulate protease, lipases, chitinases and some other exoenzymes production [29, 30]. Quorum sensing assay performed with the indicator strain *C. violaceum* CV026 revealed clear short chain AHL production in *C. fungivorans* Te331, Ter6 and *C. pratensis* Ter91, Ter291 strains, but no or trace amounts in *C. arenae* Ter10 and Ter282 strains (Additional file 1: Figure S3B). In all strains, AHL production was detected when *A. tumefaciens* NT1 was used as QS bioreporter (Additional file 1: Figure S3C).

### Secondary metabolome of *Collimonas* strains

Bacteria often produce a set of secondary metabolites with antimicrobial properties important for competition and survival in competitive environments. In *Collimonas*, the secondary metabolites are thought to play an important role enabling mycophagous growth, namely by disturbing the fungal membrane integrity [2]. Although *Collimonas* was suggested to represent a valuable resource for the discovery of novel molecules and enzymes [2], to date only two antimicrobial compounds were described for this genus, namely violacein and collimomycin [31, 32]. Violacein was identified in the *Collimonas* CT strain isolated from an aquatic environment and revealed antibacterial activity against *Micrococcus luteus* [31]. However, in the six *Collimonas* genomes here, no genes encoding violacein were identified.

Collimomycin is a polyacetylenic compound with alternating triple and single carbon-carbon bonds [32] which is produced by *C. fungivorans* Ter331 and was shown to have strong antifungal activities [32]. The corresponding biosynthesis cluster K, is only partially present in *C. fungivorans* Ter6, and completely absent in the other four genomes (Fig. 3c, Additional file 2: Table S5). Moreover, the part of the collimomycin gene cluster that is present only in *C. fungivorans* Ter331 is located in a genomic island suggesting that its presence is due to a horizontal gene transfer event.

#### Exoenzymes

Exoenzymes are extracellular enzymes produced inside the cell, and subsequently released outside the cell to perform extracellular digestion. Exoenzymes may be of importance for *Collimonas* nutrient acquisition, microbial interactions, mycophagy and weathering.

##### Chitinases

Chitin is a major component of fungal cell walls and is a homopolymer of *N*-acetyl-D-glucosamine (GlcNAc). Chitinases are able to hydrolyze the 1,4-beta-linkages of chitin [33]. Two loci A and B of chitinase biosynthesis and transport were already found in the genome of *C. fungivorans* Ter331 [34]. We observed that the other five *Collimonas* genomes also contain a complete set of genes in these two loci (Additional file 2: Table S6). This is in line with previous observations based on comparative genomic hybridization study [7] and indicates that acquisition of these genes occurred before *Collimonas* speciation. Phenotypic evaluation of chitinase production of these strains was confirmed by halo formation on water-agar plates containing colloidal chitin [1]. Bacterial chitinase activity has often been reported to be linked to antifungal properties [35–38]. Indeed, when adding chitinase inhibitor allosamidin, the growth of *Collimonas* on fungi was decreased, suggesting potential contribution to its mycophagous ability [39]. However, mutants in the chitinase loci of *C. fungivorans* Ter331 showed no difference during in vitro antagonism tests [34]. This suggests that chitinases might not contribute solely to the antifungal activities of *Collimonas*. The antifugal activities might be coupled with the production of other secondary metabolites.

##### Phospholipases

**Fig. 4:**
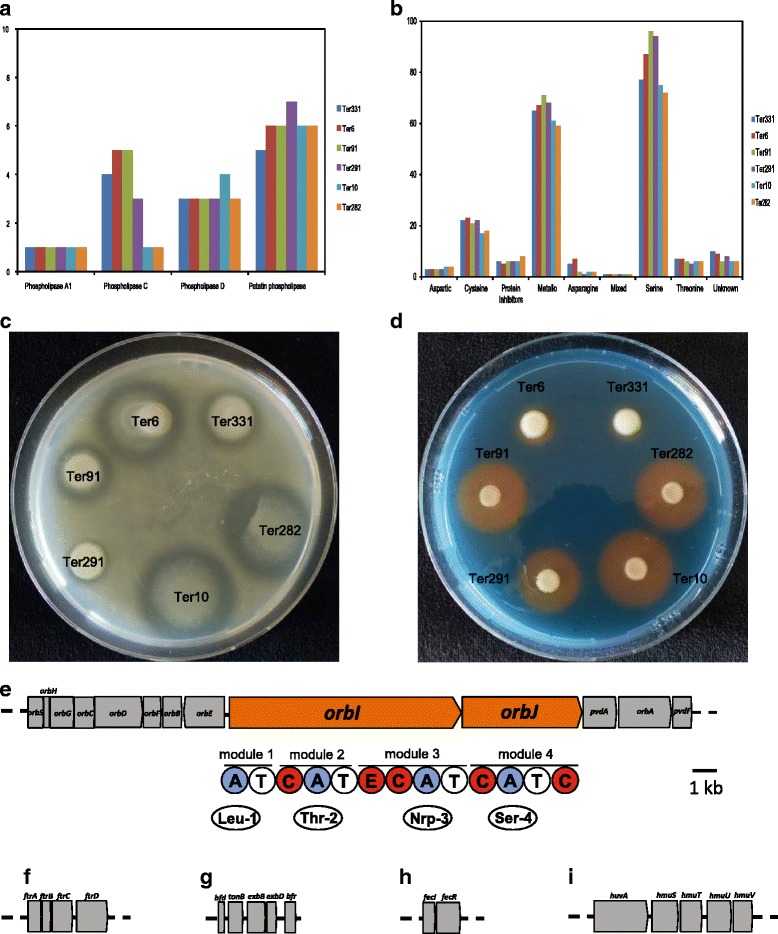
**a** Identification of phospholilpases in the six *Collimonas* genomes with PFAM signatures of the different phospholipase groups. **b** Identification of peptidases in the six *Collimonas* genomes as inferred from MEROPS 9.12 database. **c** Extracellular protease activity of *Collimonas* strains Ter331, Ter6, Ter91, Ter291, Ter10 and Ter282. A halo indicates extracellular protease production. **d** Siderophore production of *Collimonas* strains Ter331, Ter6, Ter91, Ter291, Ter10 and Ter282 plated on a CAS plate. An orange halo indicates of siderophore production. Gene clusters involved in iron acquisition in *Collimonas* strains. Five loci are presented: (**e**) Ornibactin locus. Underneath the genes are the module and domain organization of *orbI* and *orbJ*. The domains are as follows: C, condensation; A, adenylation; T, thiolation; and E, Epimerization. Underneath the domains are the amino acids that are incorporated into the peptide moiety. The number associated with the amino acid refers to the position of the amino acid in the peptide chain. **f** *ftr*_*bcc*_ *ABCD* locus. **g** *bfr* (bacterioferritin) encoding gene and adjacent genes *bfd* and *tonB*-*exbB*-*exbD* cluster. **h** *fecIR* operon. **i** *hmu* operon (or *bhu Burkholderia* haem uptake operon)

Phospholipases are a group of enzymes that catalyze the cleavage of phospholipids. Two major phospholipase activities can be defined by the site of cleavage, namely in the hydrophobic diacylglycerol moiety (PLA) or in the polar head group of the amphipathic phospholipid (PLC and PLD) (Schmmiel and Miller, 1999). In general, 11 to 15 phospholipases from four different groups: phospholipase A1, phospholipase C, phospholipase D and patatin phospholipase were detected in the six *Collimonas* genomes (Fig. 4a; Additional file 2: Table S7). Phospholipases are considered virulence factors for pathogenic bacterial species which cause tissue destruction, lung infections, hemolysis etc. [40]. Given the cleavage properties of phospholipids we speculate that they might be involved in nutrient acquisition via fungal membrane disturbing activities as well as in defense against competitors.

##### Peptidases

In the six *Collimonas* genomes, 176 to 212 peptidases were predicted and nine families of proteolytic enzymes were identified (Fig. 4b). Among them, serine and metallo peptidases are the two dominant families. The *Collimonas* strains in our study were tested positive for exoprotease production (Fig. 4c). Serine proteases are one of the most abundant groups of proteolytic enzymes found in all living organisms [41] and in prokaryotes, serine proteases are involved in several biological processes associated with cell signaling, defense response and development [42–44]. Furthermore, serine protease can be involved in regulating the biosynthesis of lipopeptides, which can play a role in the suppression of other microbes [45].

#### Iron acquisition

Siderophores are low molecular weight, high-affinity iron chelating compounds produced by microorganisms under iron limited conditions and function in solubilization, transport and storage of iron [46, 47]. Siderophore production can act as an antagonistic mechanism by scavenging limited iron from the soil environment, thereby reducing the amount of available iron for other organisms. Our analysis revealed that all six strains encode biosynthesis clusters which resemble ornibactin (Fig. 4e), a siderophore synthesis cluster of *Burkholderia cenocepacia* [48]. Siderophore production was confirmed for all the six *Collimonas* strains, albeit with different production efficiency (Fig. 4d).

Next to siderophore production, other mechanisms of iron acquisition were reported. For example, recently, a novel alternative siderophore-independent iron uptake system was identified in *Burkholderia*, named *ftr*_*bcc*_*ABCD* locus. This *ftrABCD* operon was identified in all six *Collimonas* genomes (Fig. 4f), indicating that there are more strategies for iron uptake besides ornibactin production. Moreover, we found genes coding for the production of bacterioferritin, a type of iron-storage protein [49]. The gene is often adjacent to genes encoding a small [2Fe-2S]-ferredoxin Bfd. Downstream of the *bfd* gene is the *tonB*-*exbB*-*exbD* cluster which encodes a system to transduce cellular energy to outer-membrane receptors for siderophores and haemin [50]. The bacterioferritin encoding gene *bfr* and adjacent genes *bfd* and *tonB*-*exbB*-*exbD* cluster were found in the six *Collimonas* genomes (Fig. 4g). Furthermore, a *fecIR* operon was also found in all six genomes (Fig. 4h). It is known that the transcription of genes for ferric-citrate transport in *E. coli* requires FecI, and FecR, a cytoplasmic membrane protein encoded by the second gene in the Fur-repressed *fecIR* operon which transmits an external iron signal to the cytoplasmic FecI protein [51]. A haem uptake system similar to that of *Burkholderia cenocepacia* J2315 was found in the genomes of *C. fungivorans* Ter6 and *C. pratensis* Ter91, encoded by the *hmu* operon [52]. This operon is comprised of five genes (Fig. 4i) and was suggested to be Fur-mediated. Furthermore, fungi are known to produce haem [53], and, therefore, it is plausible that *Collimonas* are able to use the fungal haem as source of iron.

#### NRPS and PKS-NRPS genes encoding metabolites

##### Lipopeptides

**Fig. 5:**
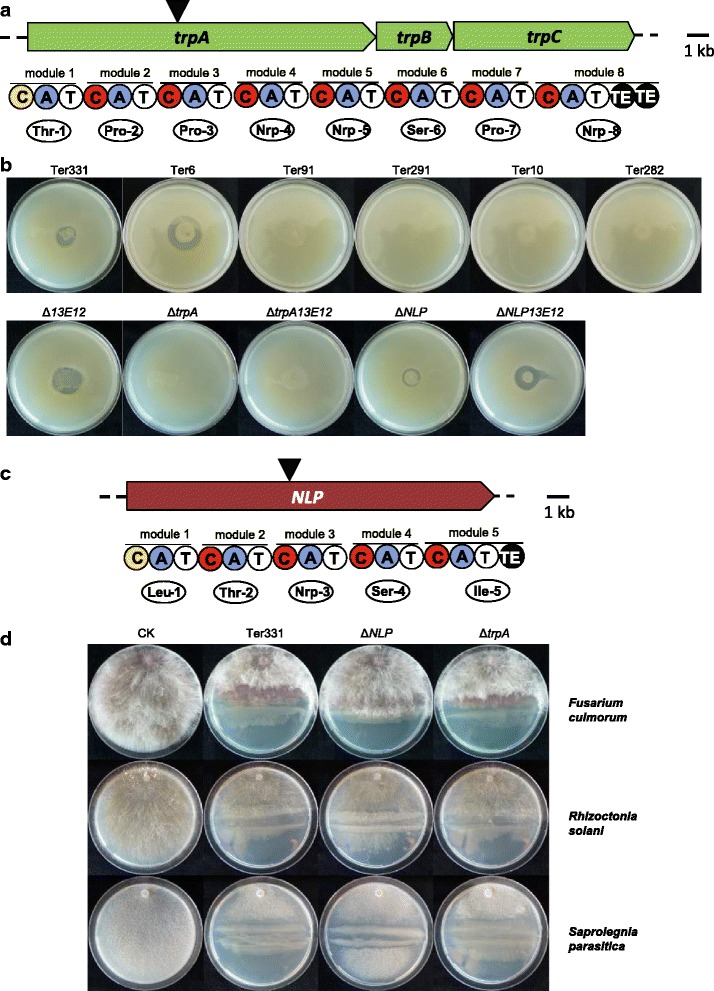
Biosynthetic gene cluster and antibacterial activity associated with tripropeptin A production by the *Collimonas* stains. **a** Organization of the gene cluster and predicted amino acid composition of the Tripropeptin A in *C. fungivorans* Ter331 and Ter6 genomes. Underneath the genes are the module and domain organization of *trpA*, *trpB* and *trpC*. The domains are as follows: C, condensation; A, adenylation; T, thiolation; and TE, thioesterification. Underneath the domains are the amino acids that are incorporated into the LP peptide moiety. The number associated with the amino acid refers to the position of the amino acid in the LP peptide chain. The black triangle indicates the position of the Gm cassette insertion in the *trpA* gene. **b** Antibacterial activity associated with tripropeptin A production. Strains Ter331, Ter6, Δ13E12 mutant (deficient in collimomycin biosynthesis), ΔNLP mutant (deficient in the new lipopeptide biosynthesis) and ΔNLP13E12 mutant (deficient in both-new lipopeptide and collimomycin biosynthesis) exhibited inhibition against *Staphylococcus aureus* via overlay assay. Δ*trpA* mutant of *C. fungivorans* Ter331 (deficient in tripropeptin A biosynthesis)- loss in the inhibition activity. Biosynthetic gene cluster and antifungal/oomycetal activities associated with new lipopeptide production by the *Collimonas* stains. **c** Organization of the gene cluster and predicted amino acid composition of the new lipopeptide in *C. fungivorans* Ter331, Ter6 and *C. pratensis* Ter91, Ter291 genomes. Underneath the genes are the module and domain organization of *NLP*. The domains are as follows: C, condensation; A, adenylation; T, thiolation; and TE, thioesterification. Underneath the domains are the amino acids that are incorporated into the LP peptide moiety. The number associated with the amino acid refers to the position of the amino acid in the LP peptide chain. The black triangle indicates the position of the Gm cassette insertion in the *nlp* gene. **d** Antifungal/oomycetal activities associated with new lipopeptide production. Strain Ter331 which has new lipopeptide biosynthetic clusters exhibited inhibition against *Fusarium culmorum*, *Rhizoctonia solani* and *Saprolegnia parasitica*. Mutant Δ*NLP* of Ter331 deficient in new lipopeptide biosynthesis abolished the inhibition activities. CK is control grew without bacteria

Lipopeptides (LPs) are compounds composed of a lipid tail with a linear or cyclic oligopeptide [54]. They exhibit surfactant, antimicrobial, anti-predation, and cytotoxic properties [55, 56]. LPs are synthesized in bacteria by large nonribosomal peptide synthetases (NRPSs) via a thiotemplate process. The structural diversity of the LPs is due to differences in the length, composition of the fatty acid tail, and the number, type and configuration of the amino acids in the peptide moiety. Via *in silico* analysis, we identified gene clusters for LP biosynthesis of tripropeptin A in the genomes of *C. fungivorans* Ter331 and Ter6 (Fig. 5a). Although the structure of tripropeptin A has been known for more than a decade [57, 58], genetic analysis of its biosynthesis was only recently reported [59]. Our study revealed that three NRPS genes *trpA*, *trpB*, *trpC* are organized in a single-operon (Fig. 5a). The *trpA* gene, encodes an NRPS with the first five modules, and *trpB*, *trpC* encode one and two modular NRPS, respectively. The wild type *C. fungivorans* Ter331 and Ter6 possess antibacterial activity against *Staphylococcus aureus* but the other four strains from *C. pratensis* and *C. arenae* (Fig. 5b) do not. The site-directed ∆*trpA* mutant of *C. fungivorans* Ter331 lacks this antagonism (Fig. 5b) indicating that tripropeptin A is indeed involved in antibacterial activity which is a unique trait for *C. fungivorans* strains.

Another 16.9 kb NRPS gene was found in *C. fungivorans* Ter331, Ter6 and *C. pratensis* Ter91, Ter291 strains (Figs. 3c and 5c). This gene is composed of five amino acids. There are no hits to known lipopeptides indicating a new lipopeptide. *Collimonas* strains are well known for their ability to suppress a range of fungi and oomycetes [3, 32, 60, 61]. However, our study revealed that the six *Collimonas* strains differed in the in vitro suppression against fungal and oomycetal pathogens (Fig. 5d; Additional file 2: Table S8). When the gene encoding the new lipopeptide was knocked out in *C. fungivorans* Ter331 by site-directed mutagenesis, reduced suppression was observed (Fig. 5d). This indicates that the new lipopeptide contributes to antimicrobial activity against both fungal and oomycetal pathogens. For the *C. arenae* species, no genes coding for the production of the new lipopeptide were found in the genome, but the strains showed suppressing activities against fungal and oomycetal pathogens (Additional file 2: Table S8). This indicates that apart from new lipopeptides, there may be other factor(s) involved in antimicrobial activity or that the suppression activity is due to synergistic effects rather than to a single compound. Thus, the different types of lipopeptide produced by the different *Collimonas* strains may partly explain the variability in the fungal inhibition behavior of these strains. The variability is also reflected in the unknown orphan gene clusters described below.

##### Unknown orphan NRPS and PKS-NRPS hybrid gene clusters

Within the genomes, four orphan gene clusters were identified. Two different 2-amino acids (2-aa) NRPS genes were found in Ter6 and Ter291 respectively (Additional file 2: Table S9; Additional file 1: Figure S4B, C). Next to this, a 54.5 kb unknown T3PKS-NRPS gene cluster was discovered in the genome of Ter6 (Additional file 1: Figure S4A). Another 10.6 kb NRPS-T1PKS gene cluster in Ter91 (Additional file 1: Figure S4D) resembles clusters encoding the Heat-stable antifungal factor (HSAF), also referred to as dihydromaltophilin, produced by *Lysobacter* species [62, 63]. HSAF exhibits inhibitory activities against a wide range of fungal species by disrupting the polarized growth or the biosynthesis of a distinct group of sphingolipids of fungi [62, 63].

#### Terpenes

**Fig. 6:**
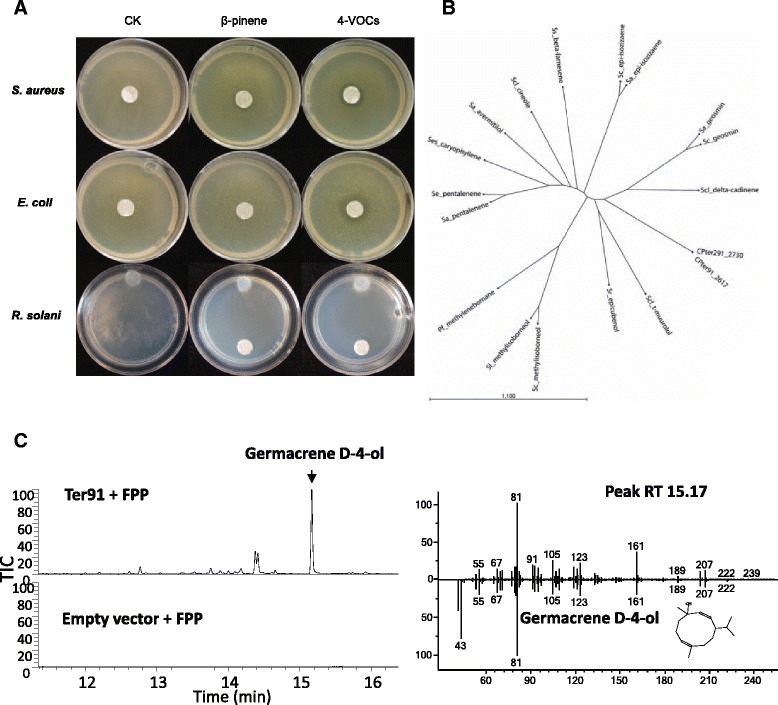
**a** Antimicrobial activities of pure β-pinene and 4-VOCs (mix of β-pinene, α-pinene, myrcene and terpinene in 1:1:1:1 ratio) against *Staphylococcus aureus*, *Escherichia coli* and *Rhizoctonia solani*. CK is control without terpenes. 5 mm sterilized white filter papers were placed in the centre or on the bottom of the petri dishes for antibacterial and antifungal assays respectively. 2 μl of each VOC was added accordingly to the white filter papers. **b** Phylogenetic tree of characterized bacterial terpene cyclase proteins. The protein name indicates the bacterial species and the major terpene produced by these terpene cyclases. Sequences included are listed in Additional file 2: Table S13. **c** Ter91 terpene synthase with FPP. TIC 100 % = 1.76E5. Major sesquiterpene product at RT 15.17, identified as Germacrene D-4-ol by comparison of mass spectra to NIST14 spectral library

Terpenes are a diverse family of primary and secondary metabolites which were mostly studied in plants and fungi [64]. Many terpenes of plant origin are known to be active against a wide variety of microorganisms, including Gram-positive, Gram-negative bacteria and fungi [65], but to date there are only few reports on antimicrobial activity of terpenes from microbial origin [66, 67]. In a previous study, four monoterpenes (γ-terpinene, 1S-α-pinene, β-pinene and β-myrcene) were detected in the headspace of *C. pratensis* strains Ter91 [68]. Here, these monoterpenes were tested individually and as a mixture for their antimicrobial activity. The β-pinene exhibited inhibition against *Staphylococcus aureus* and *Rhizoctonia solani* and the mixture of all monoterpenes revealed inhibition against these two pathogens and also to *E. coli* (Fig. 6a).

Biosynthesis of terpenes is mediated by terpene cyclases, and starts from polyprenyl pyrophosphate precursors. The precursor accepted by the cyclase determines which class of terpenes it produces: the C_10_ precursor geranyl pyrophosphate (GPP) will lead to formation of monoterpenes, while C_15_ precursorfarnesyl pyrophosphate (FPP) will lead to sesquiterpenes, and the C_20_ precursor geranylgeranyl pyrophosphate (GGPP) will lead to di-terpenes, or to phytoene (C_40_). In particular mono- and sesquiterpene synthases can occur in many cyclization patterns, leading to a huge diversity of molecules.

The genomes of the six *Collimonas* strains were screened for gene clusters possibly involved in terpene biosynthesis. All strains carry genes related to phytoene biosynthesis (Additional file 2: Table S9). Only *C. pratensis* strains Ter91 and Ter291 harbored an additional cluster comprising terpene synthases genes (Fig. 3c; CPter91_2617 and CPter291_2730). Terpene cyclases are widely distributed in bacteria, but mostly characterised in *Streptomyces* species [69, 70]. To date only one terpene cyclase from Proteobacteria has been functionally characterised, the 2-methylenebornane synthase from *Pseudomonas fluorescens* Pf0-1 [71]. CPter91_2617 and CPter291_2730 both encode a 330-amino acid protein that differs only in 2 amino acid residues (Additional file 1: Figure S5). When compared to other functionally characterized terpene cyclases, the *Collimonas* protein sequences showed maximally 23 % aa-identity to any previously characterized bacterial terpene cyclase (Fig. 6b).

The product specificity of mono- and sesquiterpene cyclases cannot be predicted from their primary sequence. For biochemical characterization, CPter91_2617 and CPter291-2730 genes were expressed in *E. coli*, partially purified and tested in vitro using FPP, GPP or GGPP as substrates. When the produced terpenes were analyzed by GC-MS, both *Collimonas* enzymes converted FPP to a mix of sesquiterpenes and sesquiterpene alcohols. The major peak was putatively identified as germacrene D-4-ol by comparison of the mass spectrum to the NIST 2014 spectral library, and several minor sesquiterpene peaks, including δ-cadinene (Fig. 6c). When GPP was applied as a substrate, the production of two monoterpenes identified as β-pinene and β-linalool was observed (Additional file 1: Figure S6A). A small amount of product could be observed upon the incubation of GGPP as substrate, which was putatively identified as 13-epimanool (Additional file 1: Figure S6B). Thus we characterized CPter91_2617 and CPter291_2730 as mixed mono-, sesqui- and diterpene cyclases, with major product germacrene D-4-ol. The sesquiterpene products suggest that they are functionally related to plant and fungal cadinene/cadinol and germacrene D-4-ol synthases, although sequence homology to these enzymes is low [72, 73]. One of the monoterpene products of CPter91_2617 and CPter291_2730, β-pinene, was also observed in the headspace of *C. pratensis* and showed antibacterial activity, suggesting a role of the *Collimonas* terpene cyclases in antimicrobial activity. Volatiles terpenes may have synergistic antimicrobial effects in combination with antibiotics. For example a synergistic effect of terpenes and penicillin on multiresistant strains *S. aureus* and *E. coli* was reported [74]. Beside antimicrobial activity the production of terpenes by *Collimonas* may point to another important ecological role, namely chemical communication. Since terpenes volatilize easily and can be produced by all kingdoms of life including plant, fungi and bacteria, we assume that terpenes may play a significant role for *Collimonas* long-distance inter-kingdom interactions and communication.

## Conclusions

The comparative analysis of six completely sequenced *Collimonas* genomes representing three species revealed a high degree of genomic diversity between strains with a core genome representing 43.1 to 52.7 % of the genome of all strains (Summarized in Fig. 3c). Although the genomes were largely syntenic, genome rearrangements were observed both between and within the species indicating high genomic plasticity. All *Collimonas* genomes carry large numbers of Genomic Islands pointing to a complex history of gene recombination and horizontal transfer between bacterial relatives. Type two secretion systems were present in all genomes suggesting that it may be important trait for the mycophagous lifestyle of *Collimonas*.

Genomic analysis of secondary metabolism of *Collimonas* revealed that genes encoding for exoenzymes such as chitinase, peptidase, phospholipase are well conserved but that there is a high variability of genes encoding for the production of other secondary metabolites such as collimomycin, lipopeptides, (PKS-)NRPS, and terpenes. Genes encoding the production of the polyacetylenic compound collimomycin and lipopeptide tripropeptin A are present only in *C. fungivorans* while the gene clusters encoding the production of a putative new lipopeptide is present in both *C. fungivorans* and *C. pratensis*, but not *C. arenae*. Moreover, several unknown orphan (PKS-)NRPS gene clusters are present in the genome of *C. fungivorans* and *C. pratensis*.

Mutational and phenotypical analyses indicated that tripropeptin A and the designated new lipopeptide have antibacterial and antifungal (oomycetal) activities, respectively. The biochemical characterization of the terpene synthases genes revealed that *Collimonas* are able to produce a set of sesquiterpenes and/or monoterpens that are considered to be mainly of plant origin. The in vitro assay of pure terpene compounds indicated their contributions to both antibacterial and antifungal activities.

Overall, our exploration of *Collimonas* genomes revealed that this bacterial group represents a valuable resource for the discovery of novel secondary metabolites and enzymes. The results gained here will be certainly helpful for designing future experimental studies that will lead to comprehensive understanding of the unique ecology of *Collimonas* species.

## Methods

### Strains and growth conditions

All bacterial strains used in this study are listed in Table 1 and Additional file 2: Table S10. *Collimonas* strains were cultured in 0.1 Tryptic Soy Broth (0.1 TSB) (5 g/L NaCl, 1 g/L KH_2_PO4, 3 g/L TSB, 20 gL − 1 CMN-Boom Agar, pH = 6.7) or King’s B medium (20 g/L proteose peptone, 1.5 g/L MgSO_4_, 1.2 g/L KH_2_PO_4_, 10 g/L glycerol, 15 g/L agar). *Escherichia coli* strain DH5α was used as a host for the plasmids used for site-directed mutagenesis. *E. coli* strains were grown on Luria-Bertani (LB) plates (10 g/L NaCl, 10 g/L Bacto™ Tryptone, 5 g/L Bacto™ Yeast extract, 20 g/L Merck Agar) or in LB broth amended with the appropriate antibiotics.

### Genomic DNA isolation

Genomic DNA from each *Collimonas* strain was extracted from overnight grown cells using QIAamp® DNA Mini Kit and Qiagen® MagAttract® HMW kit and used for Illumina and PacBio RS II sequencing respectively.

### Genome sequencing

Illumina paired-end sequences were obtained for *C. arenae* Ter10, *C. arenae* Ter282, *C. pratensis* Ter91, *C. pratensis* Ter291 *and C. fungivorans* Ter6 from BaseClear B.V. on the Illumina HiSeq2000 platform (2 x 51 bp paired-end reads, except strain Ter91 which was sequenced at 2 x 100 bp paired-end reads) and assembled using the Ray [75] assembler version 2.3.1 (Additional file 2: Table S11).

PacBio RS II sequences were obtained from 5 SMRT cells, one for each strain. Sequences were filtered using SMRT Analysis server v2.2.0 with default settings. The RS_HGAP Assembly.3 (HGAP3) [76] protocol was used to assemble the filtered reads, the RS_AH_Scaffolding protocol was used if the initial assembly yielded more than one contig, followed by a final Quiver correction using the RS_Resequencing protocol (See Additional file 2: Table S12).

The singular contigs were checked using a custom script and overlapping ends trimmed. The final circular contig for each chromosome was rearranged to start at the *dnaA* gene in the forward direction. There was no evidence of plasmids in the sequence data.

All *Collimonas* sequences including the previously published genome of Ter331 were annotated using the IGS annotation pipeline [77]. The IGS annotation of Ter331 is attached as a Additional file 3: The accession numbers of the other five genomes after submission to NCBI are Ter6 (CP013232); Ter91 (CP013234); Ter291 (CP013236); Ter10 (CP013233) and Ter282 (CP013235).

### Bioinformatic analysis

#### Core, pan and variable genome analysis

Protein coding genes from the six *Collimonas* strains were clustered together with the protein coding genes of *Burkholderia phytofirmans* PsJN (NC_010681.1, NC_010676.1) and *Pseudomonas protegens* Pf-5 (NC_004129.6) using cd-hit [78] with word length 3 (−n 3), global identity (−G 1) and a minimal alignment coverage of 60 % for the shortest protein (−aS 0.6). Cd-hit clusters were parsed into an absence-presence matrix from which the core, pan and variable genomes were parsed using custom scripts. COG annotations were determined using kognitor [79]. Core and pan evolution plot is generated based on [80]. At each number of strains (n) out of strain set (s), s!/n!*(s-n)! combinations are possible. The median number of specific, variable and core genes for all combinations are plotted as a function of n. Clusters containing one gene per strain were selected from the core cluster set of the *Collimonas*, *Burkholderia and Pseudomonas* strains were aligned with MAFFT and combined in one pseudoalignment. Redundant colums were removed and maximum likelihood phylogenetic trees were calculated with RAxML [81].

The annotation of the previously-published genome of Ter331 was updated and manually curated as part of this study. Synteny analyses were performed using Progressive MAUVE [82]. Phylogenetic analyses on the whole genome was performed using Gegenees [83], and 16S rDNA analysis was performed using MEGA6 [84–87]. Whole genome peptidases prediction was conducted by MEROPS [88]. Phospholipases were predicted by searching on the basis of the profile HMM using PFAM domains of phospholipase A1 (PF02253), phospholipase A2 (PF09056), phospholipase C (PF05506) and phospholipase D (PF00614). Secondary metabolite production clusters were examined using the antiSMASH program [89, 90]. The amino acid composition of products from NRPS sequences were predicted using NRPSpredictor 2 [91]. Genomic islands were identified using IslandViewer [92, 93] and phages elements and features were identified using PHAST [94]. CRISPRs were identified based on CRISPRfinder [10]. Whole genome analysis for type VI secretion system was conducted with SecRet6 [95] and circular genome diagrams were visualized using Circos [96].

### Availability of supporting data

Further methodological details are provided in the Additional file 4: Materials and Methods.

## Additional files

Additional file 1: Figure S1. Phylogenetic tree based on 16S rDNA depicting the relationships of sequenced strains of *Collimonas*. Underneath the genes are the module and domain organization of PKS or NRPS genes. The domains are as follows: C, condensation; A, adenylation; T, thiolation; E, Epimerization; TE, thioesterification; CAL, Co-enzyme A ligase domain; KS, Ketosynthase domain; AT, Acyltransferase domain; ER, Enoylreductase domain; KR, Ketoreductase domain; DH, Dehydratase domain; and ACP, Acyl-carrier protein domain. Underneath the domains are the amino acids that are incorporated into the peptide moiety. The number associated with the amino acid refers to the position of the amino acid in the peptide chain. Figure S2. Synteny of the six *Collimonas* genomes. Pairwise alignments of genomes were generated using Mauve (A) *C. fungivorans* (B) *C. pratensis* (C) *C. arenae* and (D) The three species together. Colored outlined blocks surround the regions of the genomic sequence that aligned to another genome. The colored bars inside the blocks are related to the level of sequence similarities. The analysis showed that the highest number of rearrangements was evident between all the three species. Figure S3. (A) Conserved gene clusters for type II (T2SSa/T2SSb), III (T3SSa/T3SSb) and VI (T6SS) secretion systems identified in *Collimonas* strains. Quorum sensing assays of the *Collimonas* strains. Quorum sensing activity of *Collimonas* strains Ter331, Ter6, Ter91, Ter291, Ter10 and Ter282 with indicator strain (B) *C. violaceum* CV026 and (C) *A. tumefaciens* NT1 (outer colonies). A purple (*C. violaceum* CV026) or blue (*A. tumefaciens* NT1) pigment produced by the indicator strains is indicative of quorum sensing activity of the tested strains. Figure S4. Organization of the orphan gene clusters and predicted amino acid compositions. Figure S5. Amino acid alignment of *Collimonas* terpene synthases CPter91_2617 and CPter291_2730 with previously characterised bacterial terpene synthases. The *Collimonas* terpene synthases were aligned with the *Streptomyces exfoliatus* pentalenene synthase (Se_pentalenene), *S. coelicolor* geosmin synthase (Sc_geosmin, 336 amino acids of the N-terminus), *Streptosporangium roseum epi*-cubenol synthase (Sr_epicubenol), *S. avermitilis* avermitilol synthase (Sa_avermitilol), *S. clavuligerus* 1,8-cineole synthase (Scl_cineole) and *Pseudomonas fluorescens* 2-methylenebornane synthase (Pf_methylenebornane). The characteristic terpene synthase divalent metal-binding motifs, namely the acidic amino acid-rich motif and the NSE triad, are boxed. Figure S6. GC-MS chromatograms of Ter91 terpene cyclase incubated with different substrates, and mass spectra of major products. The GC-MS chromatograms of Ter291 were identical to Ter91 (data not shown). Empty vector chromatograms shows products from a control enzyme extract (pACYC-duet-1). (A) Ter91 terpene synthase with GPP. TIC 100 % = 1.19E4. Major monoterpene product at RT 6.79, identified as Beta-pinene by authentic standard. (B) Ter91 terpene synthase with GGPP. TIC 100 % = 2.12E4. Major diterpene product at RT 20.24, identified as 13-epimanool comparison of mass spectra to the NIST8 library. (PDF 570 kb) Additional file 2: Table S1. Gene locus of the flagellar biosynthetic gene cluster of the *Collimonas* strains. **Table S2.** Gene locus of the secretion systems of the *Collimonas* strains. **Table S3.** Genomic distribution of signal transduction proteins in six *Collimonas* genomes. **Table S4.** Presence of quorum-sensing proteins in six *Collimonas* genomes. **Table S5.** Gene locus of the collimomycin biosynthetic gene cluster of the *Collimonas* strains. **Table S6.** Gene locus of the chitinase biosynthetic gene clusters of the *Collimonas* strains. **Table S7.** Gene locus of the phopholipase genes of the *Collimonas* strains.** Table S8.** Antimicrobial activities of the *Collimonas* strains. **Table S9.** Genes encoding secondary metabolites and secretion systems in *Collimonas* strains. **Table S10.** Strains and mutants used in this study. **Table S11.** Summary of Illumina paired-end sequences assembly data.** Table S12.** Summary of PacBio sequences assembly data. **Table S13.** Sequences of characterized bacterial terpene synthases used in the phylogenetic analysis. (XLSX 56 kb) Additional file 3:
**The IGS annotation of** ***Collimonas fungivorans*** **Ter331 genome.** (GBF 10511 kb) Additional file 4:
**Materials and Methods.** Additional detailed materials and methods. (DOCX 45 kb)

## Abbreviations

PKS: Polyketide synthase
NRPS: Nonribosomal peptide synthetase
COG: Clusters of Orthologous Groups
GIs: Genomic islands
CRISPRs: Clustered Regularly Interspaced Short Palindromic Repeats
