# Supplementary material for: Exploring the genomic traits of fungus-feeding bacterial genus Collimonas
Source: BMC Genomics. 2015 Dec 24;16:1103. doi: 10.1186/s12864-015-2289-3 (PMC4690342; doi:10.1186/s12864-015-2289-3)
Supplement: Additional file 1: Figure S1. — Phylogenetic tree based on 16S rDNA depicting the relationships of sequenced strains of Collimonas. Underneath the genes are the module and domain organization of PKS or NRPS genes. The domains are as follows: C, condensation; A, adenylation; T, thiolation; E, Epimerization; TE, thioesterification; CAL, Co-enzyme A ligase domain; KS, Ketosynthase domain; AT, Acyltransferase domain; ER, Enoylreductase domain; KR, Ketoreductase domain; DH, Dehydratase domain; and ACP, Acyl-carrier protein domain. Underneath the domains are the amino acids that are incorporated into the peptide moiety. The number associated with the amino acid refers to the position of the amino acid in the peptide chain. Figure S2. Synteny of the six Collimonas genomes. Pairwise alignments of genomes were generated using Mauve (A) C. fungivorans (B) C. pratensis (C) C. arenae and (D) The three species together. Colored outlined blocks surround the regions of the genomic sequence that aligned to another genome. The colored bars inside the blocks are related to the level of sequence similarities. The analysis showed that the highest number of rearrangements was evident between all the three species. Figure S3. (A) Conserved gene clusters for type II (T2SSa/T2SSb), III (T3SSa/T3SSb) and VI (T6SS) secretion systems identified in Collimonas strains. Quorum sensing assays of the Collimonas strains. Quorum sensing activity of Collimonas strains Ter331, Ter6, Ter91, Ter291, Ter10 and Ter282 with indicator strain (B) C. violaceum CV026 and (C) A. tumefaciens NT1 (outer colonies). A purple (C. violaceum CV026) or blue (A. tumefaciens NT1) pigment produced by the indicator strains is indicative of quorum sensing activity of the tested strains. Figure S4. Organization of the orphan gene clusters and predicted amino acid compositions. Figure S5. Amino acid alignment of Collimonas terpene synthases CPter91_2617 and CPter291_2730 with previously characterised bacterial terpene synthases. The Collimonas terpene synth [file 12864_2015_2289_MOESM1_ESM.pdf]

Figure S1

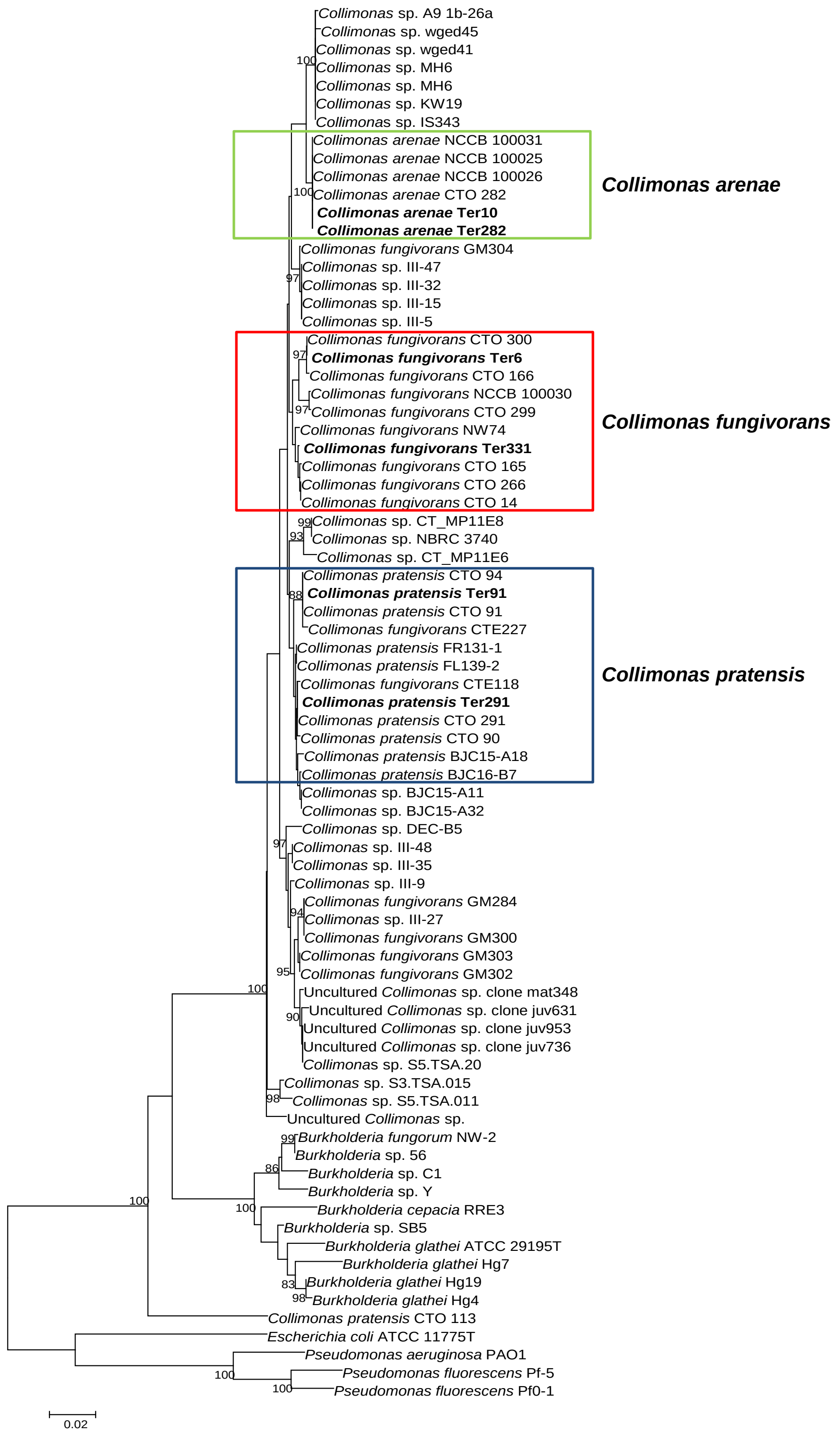
Collimonas sp. A9 1b-26a
Collimonas sp. wged45
Collimonas sp. wged41
Collimonas sp. MH6
Collimonas sp. MH6
Collimonas sp. KW19
Collimonas sp. IS343
Collimonas arenae NCCB 100031
Collimonas arenae NCCB 100025
Collimonas arenae NCCB 100026
Collimonas arenae CTO 282
Collimonas arenae Ter10
Collimonas arenae Ter282
Collimonas arenae
Collimonas fungivorans GM304
Collimonas sp. III-47
Collimonas sp. III-32
Collimonas sp. III-15
Collimonas sp. III-5
Collimonas fungivorans CTO 300
Collimonas fungivorans Ter6
Collimonas fungivorans CTO 166
Collimonas fungivorans NCCB 100030
Collimonas fungivorans CTO 299
Collimonas fungivorans NW74
Collimonas fungivorans Ter331
Collimonas fungivorans CTO 165
Collimonas fungivorans CTO 266
Collimonas fungivorans CTO 14
Collimonas fungivorans
Collimonas sp. CT_MP11E8
Collimonas sp. NBRC 3740
Collimonas sp. CT_MP11E6
Collimonas pratensis CTO 94
Collimonas pratensis Ter91
Collimonas pratensis CTO 91
Collimonas fungivorans CTE227
Collimonas pratensis FR131-1
Collimonas pratensis FL139-2
Collimonas fungivorans CTE118
Collimonas pratensis Ter291
Collimonas pratensis CTO 291
Collimonas pratensis CTO 90
Collimonas pratensis BJC15-A18
Collimonas pratensis BJC16-B7
Collimonas pratensis
Collimonas sp. BJC15-A11
Collimonas sp. BJC15-A32
Collimonas sp. DEC-B5
Collimonas sp. III-48
Collimonas sp. III-35
Collimonas sp. III-9
Collimonas fungivorans GM284
Collimonas sp. III-27
Collimonas fungivorans GM300
Collimonas fungivorans GM303
Collimonas fungivorans GM302
Uncultured Collimonas sp. clone mat348
Uncultured Collimonas sp. clone juv631
Uncultured Collimonas sp. clone juv953
Uncultured Collimonas sp. clone juv736
Collimonas sp. S5.TSA.20
Collimonas sp. S3.TSA.015
Collimonas sp. S5.TSA.011
Uncultured Collimonas sp.
Burkholderia fungorum NW-2
Burkholderia sp. 56
Burkholderia sp. C1
Burkholderia sp. Y
Burkholderia cepacia RRE3
Burkholderia sp. SB5
Burkholderia glathei ATCC 29195T
Burkholderia glathei Hg7
Burkholderia glathei Hg19
Burkholderia glathei Hg4
Collimonas pratensis CTO 113
Escherichia coli ATCC 11775T
Pseudomonas aeruginosa PAO1
Pseudomonas fluorescens Pf-5
Pseudomonas fluorescens Pf0-1
100
100
97
97
97
99
93
88
97
94
95
100
90
98
99
86
100
100
83
98
100
100
0.02

Figure S2

A

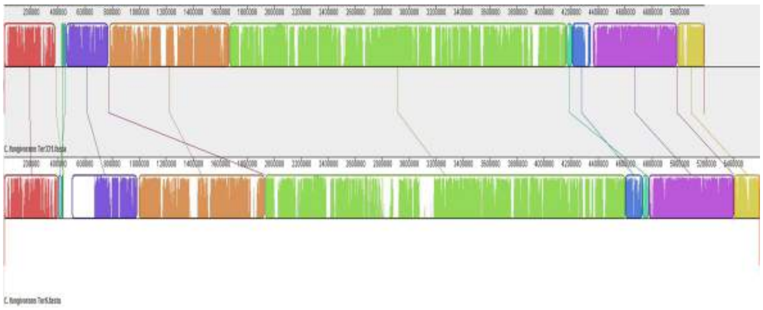

B

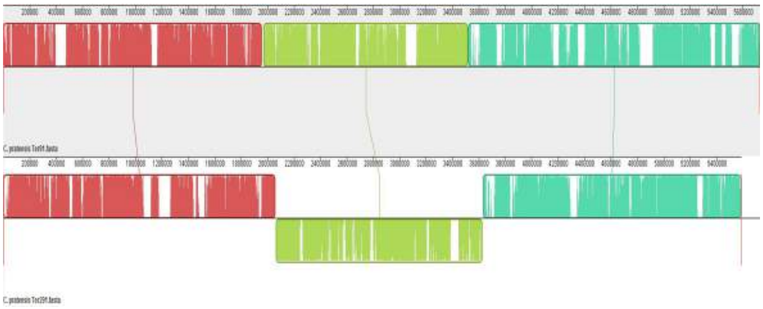

C

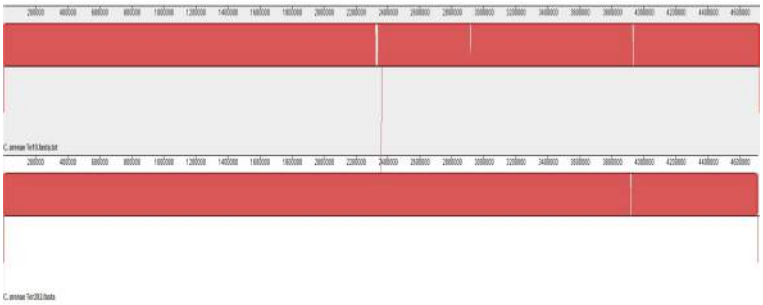

D

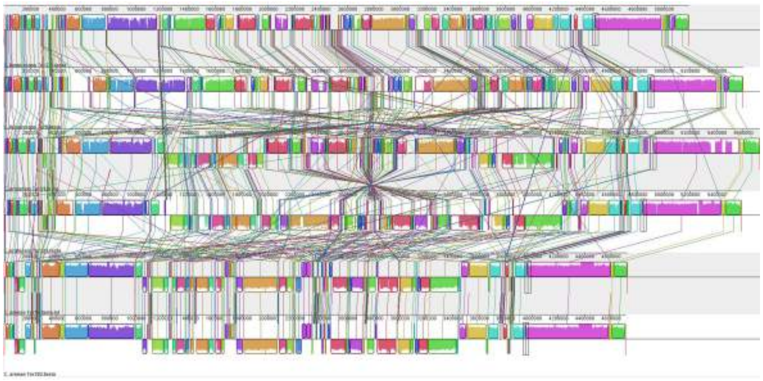

Figure S3

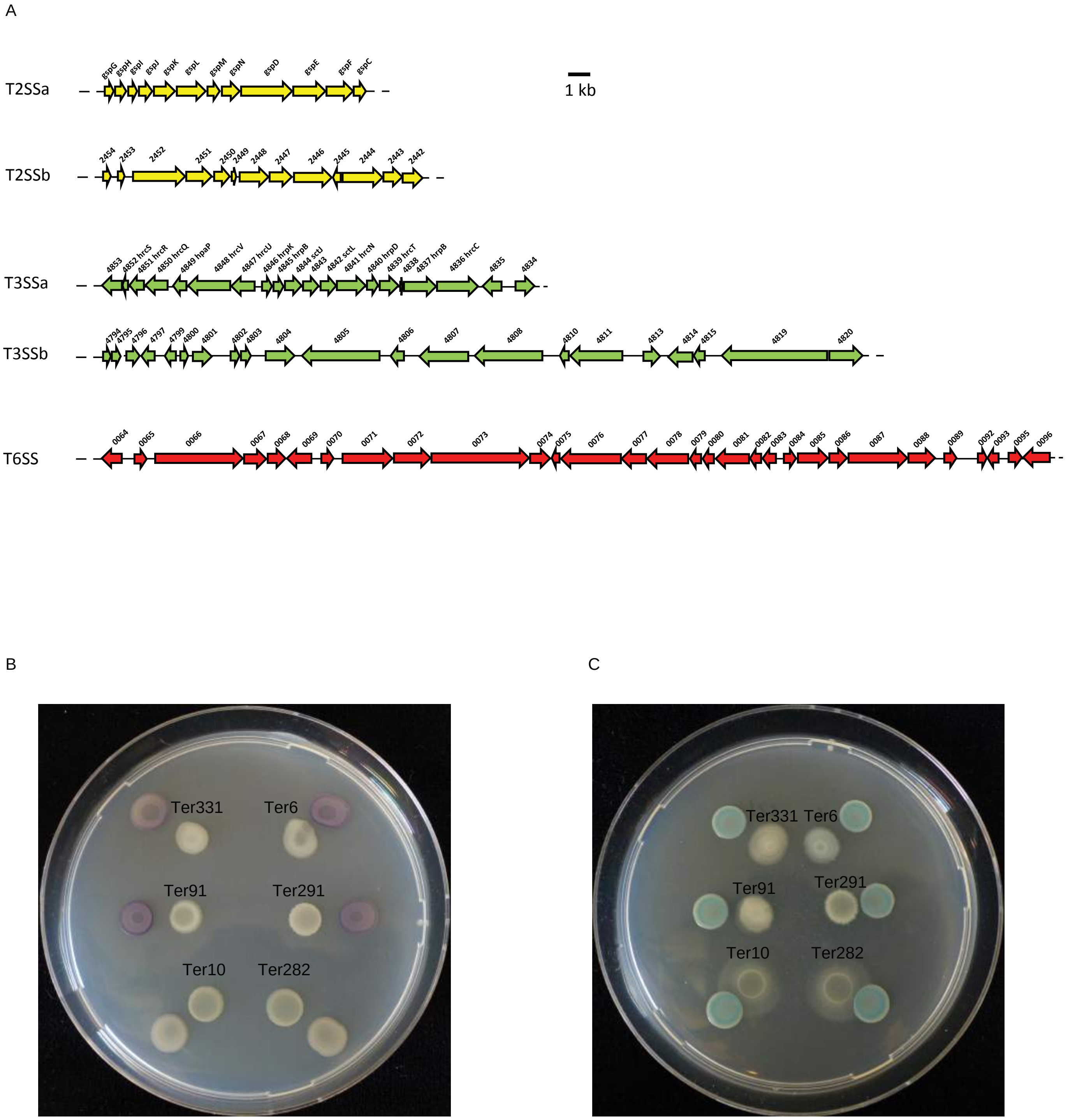
A
T2SSa
1 kb
T2SSb
T3SSa
T3SSb
T6SS
B
Ter331
Ter6
Ter91
Ter291
Ter10
Ter282
C
Ter331 Ter6
Ter91
Ter291
Ter10
Ter282

Figure S4

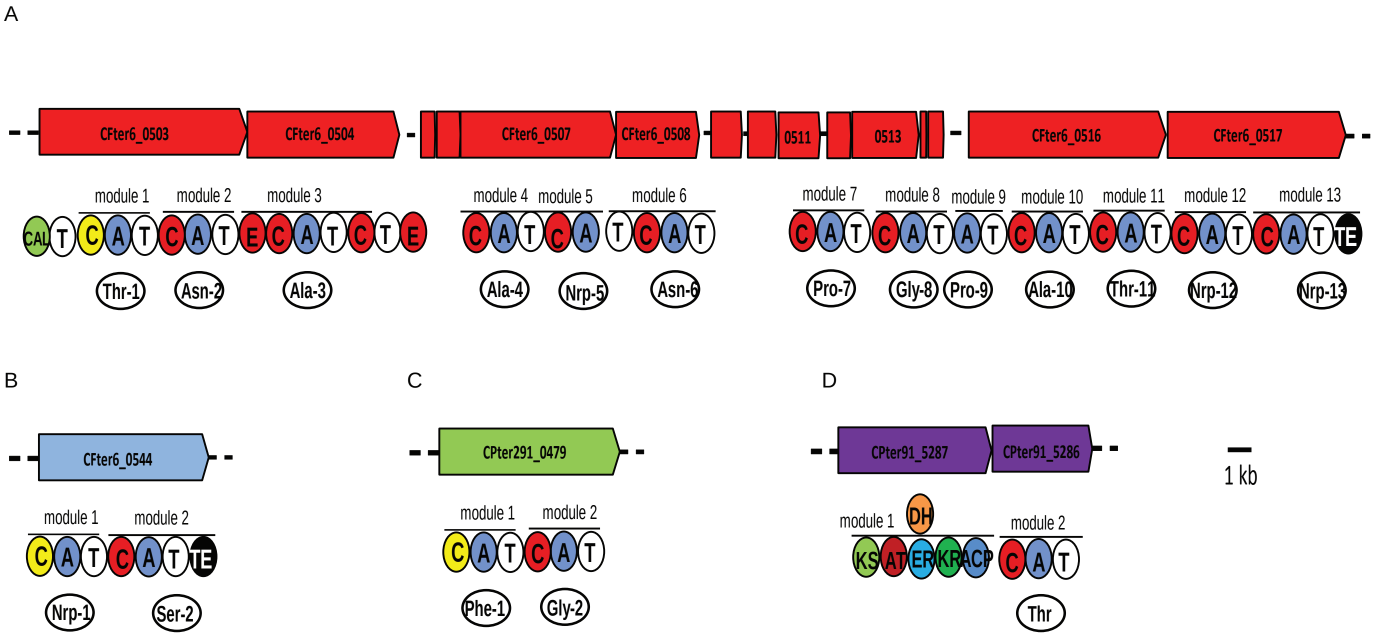
A
CFter6_0503
CFter6_0504
CFter6_0507
CFter6_0508
0511
0513
CFter6_0516
CFter6_0517
module 1
module 2
module 3
module 4
module 5
module 6
module 7
module 8
module 9
module 10
module 11
module 12
module 13
CAL T C A T C A T E C A T C T E
C A T C A T C A T
C A T C A T A T C A T C A T C A T C A T TE
Thr-1
Asn-2
Ala-3
Ala-4
Nrp-5
Asn-6
Pro-7
Gly-8
Pro-9
Ala-10
Thr-11
Nrp-12
Nrp-13
B
CFter6_0544
module 1
module 2
C A T C A T TE
Nrp-1
Ser-2
C
CPter291_0479
module 1
module 2
C A T C A T
Phe-1
Gly-2
D
CPter91_5287
CPter91_5286
module 1
DH
KS AT ER KR ACP
module 2
C A T
Thr
1 kb

Figure S5

```
                               10         20         30         40         50         60
                       ....|....| ....|....| ....|....| ....|....| ....|....| ....|....|
CPter91_2617           ---------- ---MLTFERP EFVLPFHAHT NPHQ-AIAGA NARRWLLSTG LIGADQLAQA
CPter291_2730          ---------- ---MLTFERP EFVLPFHAHT NPHQ-AIAGA NARRWLLSTG LIGADQLAQA
Se_pentalenene         ---------- -----MPQDV DFHIPLPGRQ SPDH-ARAEA EQLAWPRSLG LIRSDAAAER
Sc_geosmin_366AA       ---------- -MTQQPFQLP HFYLPHPARL NPHL-DEARA HSTTWAREMG MLEGSGVWEQ
Sr_epicubenol          ----MGTTTT HKFDRPLRLP PLPCPFPSEV NPYV-EQVDK ETLEWLIDSE MLDDAETVER
Sa_avermitilol         ---------- -----MPQDI DFGLPAPAGI SPGL-EATRR HNLGWVRRLG LVGDGPSLAW
Scl_cineole            ---------- ----MPAGHE EFDIPFPSRV NPFH-ARAED RHVAWMRAMG LITGDAAEAT
Pf_methylenebornane    MNQSSSARTP RSATAPFIVR AVRCPPPTRI DEALGQEVNE RLMEWISNIG IFAGKEEKIR

                               70         80         90        100        110        120
                       ....|....| ....|....| ....|....| ....|....| ....|....| ....|....|
CPter91_2617           G--NER-YDE LLAKMYPHAG VLE-LEVAIC WLTWYFMLDD FLDK---QDI HS-------A
CPter291_2730          G--NER-YDE LLAKMYPHAG VLE-LEVAIC WLTWYFMLDD FLDK---QDI HS-------A
Se_pentalenene         H--LRGGYAD LASRFYPHAT GAD-LDLGVD LMSWFFLFDD LFDGPRGENP ED-------T
Sc_geosmin_366AA       SDLEAHDYGL LCAYTHPDCD GPA-LSLITD WYVWVFFFDD HFLEKYKRSQ DR-------L
Sr_epicubenol          Y--RQAKYGW LSARTYPYAE HHT-LRLVSD WCVWLFAFDD AFCESDRRAA EI-------A
Sa_avermitilol         Y--TSWDMPR LAACGFPHAR GAA-LDLCAD AMAFFFVFDD QFDGPLGRDP AR-------A
Scl_cineole            Y--RRWSPAK VGARWFYLAQ GED-LDLGCD IFGWFFAYDD HFDGPTGTDP RQ-------T
Pf_methylenebornane    A----SDFGR YAMLCHADTN NPDRLLLVAQ CFAALFAVDD HYCDDQSLGG RPETVAESLS
                                                          acidic amino acid-rich motif

                              130        140        150        160        170        180
                       ....|....| ....|....| ....|....| ....|....| ....|....| ....|....|
CPter91_2617           RDTVRSITGQ IHGDTQARIE P---DGMMAI LWSALWIRIA AAASPRSQQR FLLAQRDALL
CPter291_2730          RDTVRSITGQ IHGDTQARIE P---DGMMAI LWSALWIRIA AAASPRSQQR FLLAQRDALL
Se_pentalenene         KQLTDQVAAA LDGPLPDT-- ----APPIAH GFADIWRRTC EGMTPAWCAR SARHWRNYFD
Sc_geosmin_366AA       AGKAHLDRLP LFMPLDDAAG MPEPRNPVEA GLADLWTRTV PAMSADWRRR FAVATEHLLN
Sr_epicubenol          RALPQLYAVL EDLDVGSEV- ----DDVFAK SLLEIKGRIA AYGDDEQLDR WRNVTKDYLF
Sa_avermitilol         ARVCRRLTGI VHGAGPGPG- ----ADACSA AFADVWARST DGAHPGWVAR TAHEWEYYFA
Scl_cineole            AAFVNRTVAM LDPRADPTG- ----EHPLNI AFHDLWQRES APMSPLWQRR AVDHWTQYLT
Pf_methylenebornane    FALTAIDPVY LPSPFDKELL KQQMCDPVIR GLLAYMKRVA QFCTPSQVAR VRQITIAMFV

                              190        200        210        220        230        240
                       ....|....| ....|....| ....|....| ....|....| ....|....| ....|....|
CPter91_2617           SNLQEIENRM ERRIPD-LFS FVALRRLSGG MRTVFLFNGY ADHIELPAAF ERHPLHMALS
CPter291_2730          SNLQEIENRM ERRIPD-LFS FVALRRLSGG MRTVFLFNGY ADHIELPAAF ERHPLHMALS
Se_pentalenene         GYVDEAESRF WNAPCDSAAQ YLAMRRHTIG VQPTVDLAER AGRFEVPHRV FDSAVMSAML
Sc_geosmin_366AA       ESMWELSNIN EGRVAN-PVE YIEMRRKVGG APWSAGLVEY AT-AEVPAAV AGTRPLRVLM
Sr_epicubenol          AQVWEAANRE DEVVPS-LED YIFMRRRTGA MLTVFALIDV ASGRSLSADE WRHPGMRAIT
Sa_avermitilol         AQAHEAINRL RGTPGD-MES YLQVRRGIAG TDLPLSLGER AAGITVPAAA FHSPQLRIMR
Scl_cineole            AHITEATNRT RHTSPT-IAD YLELRHRTGF MPPLLDLIER VWRAEIPAPV YTTPEVQTLL
Pf_methylenebornane    TMAAEGPWRL YGTQPT-VAE YLASR-QVNS FWPCLVLIDL IGGYEVPANT YSRPDIHHVT

                              250        260        270        280        290        300
                       ....|....| ....|....| ....|....| ....|....| ....|....| ....|....|
CPter91_2617           DAANDVACIT NDILSFDKEA ANGET-NNYV IVVQHHMSGS IRQAVDFLHH LQAARIEAYL
CPter291_2730          DAANDVACIT NDILSFDKEA ANGET-NNYV IVVQHHMRGS IRQAVDFLHH LQAARIEAYL
Se_pentalenene         QIAVDVNLLL NDIASLEKEE ARGEQ-NNMV MILRREHGWS KSRSVSHMQN EVRARLEQYL
Sc_geosmin_366AA       ETFSDAVHLR NDLFSYQREV EDEGELSNGV LVLETFFGCT TQEAADLVND VLTSRLHQFE
Sr_epicubenol          ESANDVVVWD NDLISYAKES NSGNSRNNLV NVLAEHRHYS RQEAMEEIGE MRNQAIADMV
Sa_avermitilol         EAAIDVTLMC NDVYSLEKEE ARGDM-DNLV LVIEHARRCT RDEAVTAARG EVARRVIRFE
Scl_cineole            HTTNQNINIV NDVLSLEKEE AHGDP-HNLV LVIQHERQST RQQALATARR MIDEWTDTFI
Pf_methylenebornane    ALASLATTLV NDLYSAYKEH LNETGDFKLP YLLAARHNCS LQEAIDLAAD IHDAVMEEYE
                                NSE triad

                              310        320        330        340        350        360
                       ....|....| ....|....| ....|....| ....|....| ....|....| ....|....|
CPter91_2617           HAKK-QLPQV FRQLHLPPAQ QVRAIQYLR- GVEDALSGLL DWSLQTPRYG GTAVAHRNQE
CPter291_2730          HAKK-QLPHV FRQLHLPPAQ QVRAIQYLR- GVEDALSGLL DWSLQTPRYG GTAVAHRNQE
Se_pentalenene         LLES-CLPKV GEIYQLDTAE REALERYRTD AVRTVIRGSY DWHRSSGRYD -AEFALAAGA
Sc_geosmin_366AA       HTAFTEVPAV ALEKGLTPLE VAAVGAYTK- GLQDWQSGGH EWHMRSSRYM NKGERPLAGW
Sr_epicubenol          AVRP-SLEAL G--------- SDAVLAYVR- GLEFWISGSV DYSLTSSRYT DAWRTARQPS
Sa_avermitilol         QLAR-EVPAL CAQLGLSAVE RAHVDTYLG- VMEAWMSGYH AWQTQTRRYT GAPHVLPSTG
Scl_cineole            RTEP-RLPAL CGRLGIPLAD RTSLYTAVE- GMRAAIRGNY DWCAETNRYA VHRPTGTGRA
Pf_methylenebornane    RLHATLMKGT R--------- SPVLRRYLT- GLSTWIGGNL EWHKHSARYH I---------
```

Figure S6

A

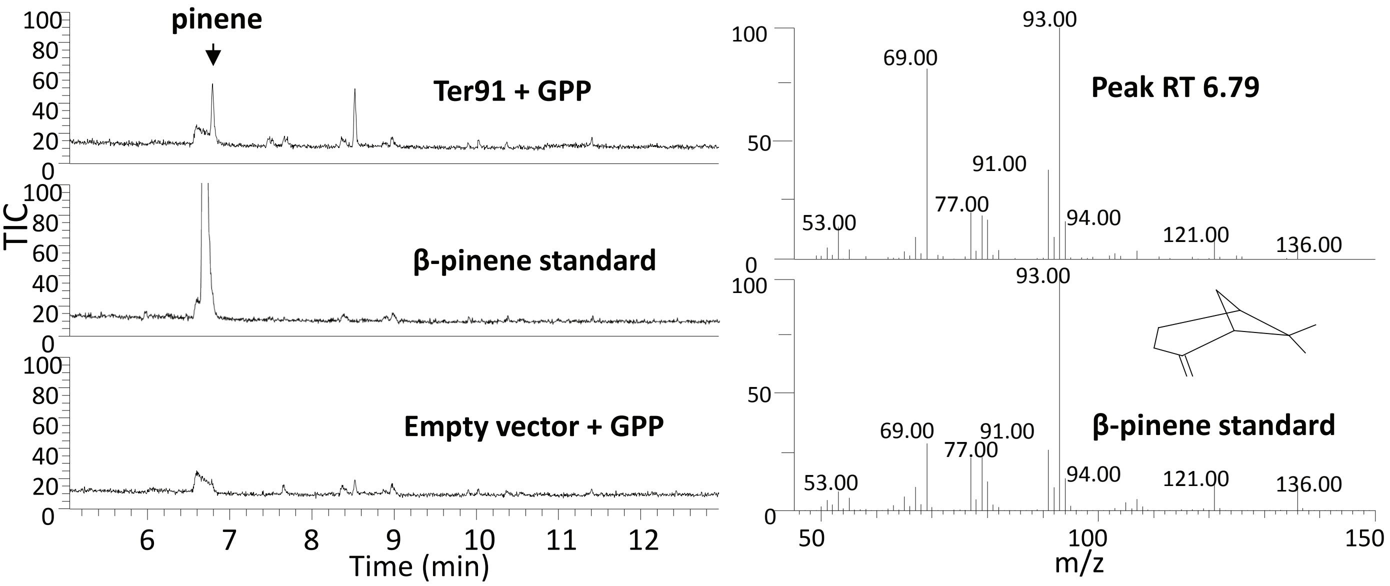

pinene
Ter91 + GPP
β-pinene standard
Empty vector + GPP
TIC
Time (min)
Peak RT 6.79
93.00
69.00
91.00
77.00
53.00
94.00
121.00
136.00
β-pinene standard
m/z

B

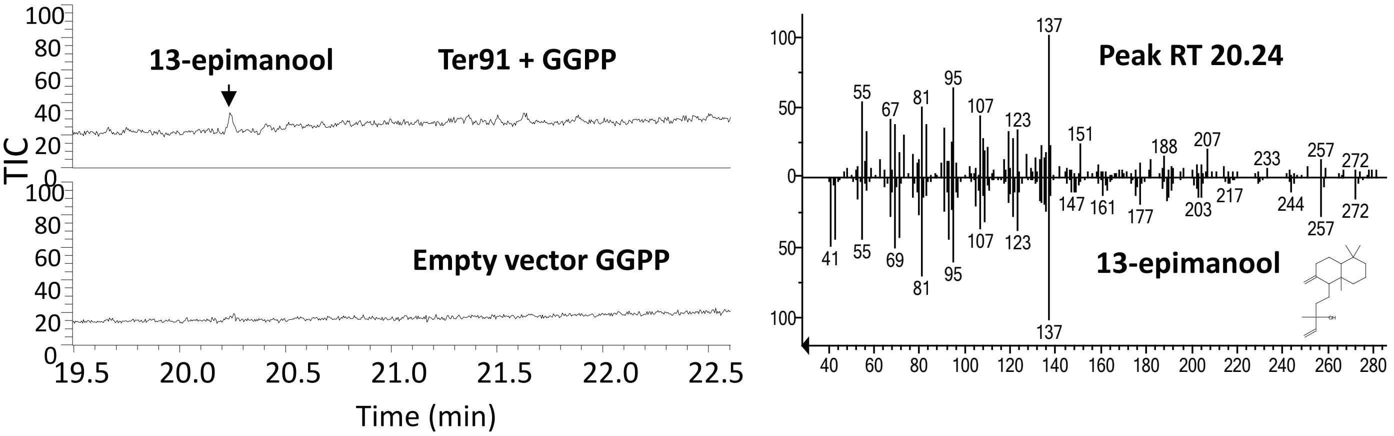

13-epimanool
Ter91 + GGPP
Empty vector GGPP
TIC
Time (min)
Peak RT 20.24
13-epimanool
