# Supplementary material for: Exploring the genomic traits of fungus-feeding bacterial genus Collimonas
Source: BMC Genomics. 2015 Dec 24;16:1103. doi: 10.1186/s12864-015-2289-3 (PMC4690342; doi:10.1186/s12864-015-2289-3)
Supplement: Additional file 4: — Materials and Methods. Additional detailed materials and methods. (DOCX 45 kb) [file 12864_2015_2289_MOESM4_ESM.docx]

**Exploring the genomic traits of fungus-feeding bacterial genus *Collimonas***

Chunxu Song†^1^, Ruth Schmidt^1^, Victor de Jager^1^, Dorota Krzyzanowska^2^, Esmer Jongedijk^3^, Katarina Cankar^3^, Jules Beekwilder^3^, Anouk van Veen^1^, Wietse de Boer^1^, Hans van Veen^1^, Paolina Garbeva^1^

^1^Netherlands Institute of Ecology, Department of Microbial Ecology, Droevendaalsesteeg 10, 6708 PB Wageningen, the Netherlands

^2^Laboratory of Biological Plant Protection, Intercollegiate Faculty of Biotechnology UG&MUG, Kladki 24, 80-822 Gdansk, Poland

^3^Business Unit Bioscience, Plant Research International, Wageningen University and Research Centre Wageningen, Netherlands

**^†^**Corresponding author: Chunxu Song

Email addresses:

CS: [c.song@nioo.knaw.nl](mailto:c.song@nioo.knaw.nl)

RS: [r.schmidt@nioo.knaw.nl](mailto:r.schmidt@nioo.knaw.nl)

VJ: [v.dejager@nioo.knaw.nl](mailto:v.dejager@nioo.knaw.nl)

DK: [dorota.krzyzanowska@biotech.ug.edu.pl](mailto:dorota.krzyzanowska@biotech.ug.edu.pl)

EJ: [esmer.jongedijk@wur.nl](mailto:esmer.jongedijk@wur.nl)

KC: [katarina.cankar@wur.nl](mailto:katarina.cankar@wur.nl)

JB: [jules.beekwilder@wur.nl](mailto:jules.beekwilder@wur.nl)

AV: [anouk_v_veen@hotmail.com](mailto:anouk_v_veen@hotmail.com)

WB: [w.deboer@nioo.knaw.nl](mailto:w.deboer@nioo.knaw.nl)

JV: [h.vanveen@nioo.knaw.nl](mailto:h.vanveen@nioo.knaw.nl)

PG: [p.garbeva@nioo.knaw.nl](mailto:p.garbeva@nioo.knaw.nl)

**Supplemental Materials and Methods**

**Quorum sensing assay**

*Collimonas* wild type strains, and indicator strain *C. violaceum* CV026 (50 μg/mL kanamycin) [[1](#_ENREF_1)], *A. tumefaciens* NT1 [[2](#_ENREF_2)] were grown in 10 ml 0.1 TSB broth overnight at 20°C. The cells were washed twice with sterile 10 mm sodium phosphate buffer (1.361g KH_2_PO_4_ in 1L milliQ, pH 6.5), adjusted to 1x10^8^ cells/ml, 5µl of cell suspension was spotted on 0.1 TSB plates with indicator strain *C. violaceum* CV026 and 0.1 TSB plates (50 μg/mL X-gal) with indicator strain *A. tumefaciens* NT1 and incubated at 20°C for 2-3 days. Purple (*C. violaceum* CV026) and blue (*A. tumefaciens* NT1) colonies are indicating production of AHLs by the respective *Collimonas* strains.

**Siderophore detection assay**

ChromoAzurolS (CAS) and King’s B (KB) medium were prepared based on previously description [[3](#_ENREF_3)]. KB medium consists of 20g proteose peptone, 1.5g MgSO_4_, 1.2g KH_2_PO_4_, 10g glycerol, 15g agar and water to 1L. Orange halos around the colonies on the blue CAS agar plates are indicative of siderophore production.

**Extracellular protease activity assay**

The cells from different strains were washed with sterile MilliQ water and set to a final density of 1x10^8^ cells/mL and 5µL of this bacterial suspension was spotted on Skim Milk Agar plates (SMA, 1 Liter: 15g skim milk powder, 4g blood agar base, 0.5g yeast extract and 13.5g agar) and incubated at 20°C for 4 days. Extracellular protease activity was quantified by measuring the diameter of the transparent halo surrounding the bacteria colony.

**Swimming motility**

Swimming motility assays of the bacterial strains were conducted according to the method described previously by de Bruijn & Raaijmakers [[4](#_ENREF_4)]. Swimming motility of the *Collimonas* strains were assessed on soft [0.3% wt/vol] M9 medium. After autoclaving, the medium was cooled down in a water bath to 55°C and kept at 55°C for 1 h. Twenty ml of the medium was pipetted into a 9-cm-diameter petri dish, and the plates were kept for 24 h at room temperature (20°C) prior to the swimming assay. For all swimming assays, the same conditions (agar temperature & volume, time period of storage of the poured plates) were kept constant to maximize reproducibility. Overnight cultures of *Collimonas* strains were washed three times with 0.9% NaCl, and 5 µL of the washed cell suspension (1x10^8^ cells/ml) was spot inoculated in the centre of the soft agar plate and incubated for 3 days at 20°C.

**Mutagenesis of new lipopeptide and tripropeptin A genes**

Site-directed mutagenesis of the new lipopeptide and tripropeptin A was performed with the pEX18Tc suicide vector as described by Choi and Schweizer [[5](#_ENREF_5)]. For each mutant construct, a 5′ fragment, a Gm cassette and a 3′ fragment were synthesized for the target gene in Baseclear, Leiden, the Netherlands ([www.baseclear.com](http://www.baseclear.com)), and constructed to vector pEX18Tc. The synthesized sequences are given in the end of this file. The mutant constructs pEX18Tc-*NLP* and pEX18Tc-*trpA* were were subsequently electroporated into *C. fungivorans* Ter331. Electrocompetent cells were obtained according to the method of Choi et al. [[6](#_ENREF_6)] and the electroporation was performed at 2.4 kV and 200 µF. After incubation in SOC medium (2% Bacto tryptone [Difco], 0.5% Bacto yeast extract [Difco], 10 mM NaCl, 2.5 mM KCl, 10mM MgCl2, 10mM MgSO4, 20mM glucose [pH 7]) for 2 h at 25°C, the cells were plated on KB supplemented with gentamicin (40µg/ml). The obtained single crossover colonies were grown in LB overnight at 25°C and plated on LB supplemented 5% sucrose and gentamicin (40µg/ml) to accomplish the double crossover. The plates were incubated at 25°C for at least 48 h, and colonies were re-streaked on KB supplemented with gentamicin (40µg/ml) and tetracycline (25µg/ml). Colonies that grew on KB with gentamicin, but not on KB with tetracycline, were selected and subjected to colony PCR to confirm genes mutagenesis.

**Phylogenetic analysis of terpene synthases**

The deducted protein sequence of terpene synthases CPter91_2617 and CPter291_2730 was compared to previously characterized terpene synthases from genera *Streptomyces, Saccharopolyspora, Saccharothrix, Streptosporangium* and *Pseudomonas*. A full list of sequences included for phylogenetic analysis is given in Supplemental table S14. Multiple protein sequence alignments and bootstrap N-J trees were generated by the CLC Workbench software, with a 100 replicates of bootstrap analysis.

**Heterologous expression of terpene synthases in *E. coli* and enzyme activity assays**

PCR-generated DNA encompassing the complete coding sequence of terpene synthases genes of CPter91_2617 and CPter291_2730 were inserted into the cloning site of the expression vector pACYCDuet-1 (Cm^R^). The constructs were introduced into *E. coli* BL21 DE3 and protein expression and enzyme activity assays were performed as described in Jongedijk et al. [[7](#_ENREF_7)]. 5 µl 10 mm GPP, FPP or GGPP were added as substrates to the assay mix. The mix was immediately covered with an overlay of 1 ml pentane and incubated at 30°C for 1 h under gentle agitation. The tubes were vortexed well and centrifuged for 5 min at 3400 rpm. The pentane phase was collected, dried over anhydrous Na_2_SO_4_ and injected into a 7890A gas chromatograph (Agilent) equipped with a mass selective detector (Model 5975C, Agilent), scanning in the range 45–450 m/z. Splitless injection of 1 μl sample was performed at 250°C on a Zebron ZB-5MS column (30m× 0.25mm, 0.25 μm thickness; Phenomenex) at a helium flow rate of 1 ml/min. The temperature programme was 2.25 min at 45°C, then the temperature was increased at the rate of 40°C/min to 300°C, followed by 3 min at 300°C. Standard of β-pinene was purchased from Acros.

**Antimicrobial activities assay**

The antifungal and anti-oomycete activities of the *Collimonas* strains and mutants were tested as follows: *Collimonas* strains and mutant strains were grown in 5 ml KB broth overnight at 25 °C. Strip 50 µl bacterial suspension (1x10^8^ cells/ml) in the middle of a 0.1 TSB plate. After three days of incubation at 20°C, a mycelial plug of 4-mm diameter of each fungal or oomycete pathogen was placed in the edge of the 0.1 TSB plate and incubated at 20°C. Migration diameters of the fungus or oomycete were measured for 6-7 days depending on the pathogen’s growth rate.

To test the antibacterial activity of *Collimonas* strains or constructed mutants, 10 µl of cell suspension was spotted on 0.1 TSB plates and incubated at 20°C for 3 days. Subsequently, overnight cultures of *S. aureus* was washed twice with sterile milliQ water, and cell suspensions (2x10^6^ cells/ml) were overlaid onto the *Collimonas*/mutant inoculated agar surface and incubated at 30⁰C overnight. The antibacterial activity was observed by the formation of visible zones of inhibition of the bacterial pathogens.

**References:**

1. McClean KH, Winson MK, Fish L, Taylor A, Chhabra SR, Camara M, Daykin M, Lamb JH, Swift S, Bycroft BW *et al*: **Quorum sensing and *Chromobacterium violaceum*: exploitation of violacein production and inhibition for the detection of N-acylhomoserine lactones**. *Microbiol-Uk* 1997, **143**:3703-3711.

2. Farrand SK, Qin YP, Oger P: **Quorum-sensing system of *Agrobacterium* plasmids: analysis and utility**. *Method Enzymol* 2002, **358**:452-484.

3. Schwyn B, Neilands JB: **Universal chemical-assay for the detection and determination of siderophores**. *Anal Biochem* 1987, **160**(1):47-56.

4. de Bruijn I, Raaijmakers JM: **Regulation of cyclic lipopeptide biosynthesis in *Pseudomonas fluorescens* by the ClpP protease**. *J Bacteriol* 2009, **191**(6):1910-1923.

5. Choi KH, Schweizer HP: **An improved method for rapid generation of unmarked *Pseudomonas aeruginosa* deletion mutants**. *BMC microbiology* 2005, **5**:30.

6. Choi KH, Kumar A, Schweizer HP: **A 10-min method for preparation of highly electrocompetent *Pseudomonas aeruginosa* cells: Application for DNA fragment transfer between chromosomes and plasmid transformation**. *J Microbiol Meth* 2006, **64**(3):391-397.

7. Jongedijk E, Cankar K, Ranzijn J, van der Krol S, Bouwmeester H, Beekwilder J: **Capturing of the monoterpene olefin limonene produced in *Saccharomyces cerevisiae***. *Yeast* 2015, **32**(1):159-171.

Synthesized sequences for the new lipopeptide and tripropeptin A. Sequences in bold represents the Gm cassette. Before and after the Gm cassette is the 5′ and 3′ fragment of the target gene respectively.

1. New lipopeptide (NLP) KO sequence

AAAAAAAATCAAGCAAGCAAGCTTcaaggcgagacagagattgcgctggcggccatctggtcggccctgctgcagatcgaacgcatcggccgccacgacaacttcttctcgctgggcggccattcgctgctcgccgtgaccctgatggaaagaatgcgccagcaaggcttgcaagccgaagtacgcgccctgttttcctccccgaccctggccggactggcggcgtctatcggcgaagaaagccgcctggtcaacgtccccgccaacctgattccatccggatgcgaaaccatcacgccggaaatgctgccgatggtgacgctgaacgacgctgaaatcgccagcgttgtcggcaatgttccaggcggcgccgccaatgtgcaggatatctatccgctggcgccgttgcaggaaggcatactgttccaccacctgatggccaaggaaggcgatccctacctgctggtgggactgaccggtttcgatacccggcagcggctggaagcatacctggcagccttgcaaggcgtgatacagcggcacgacgtgctgcgcaccgcaatcgtctgggaaggcgtgccggaaccgctgcaggtggtctggcgctcggcgccgctggtgcaggaagaactgatactcgatccggccgacggcgacgtcgcgcgccagctgcgcgcccgtttcgacccgcgccacacccgcctcgacctgacgcaggcgccgctgatgcggaccagtttcgcctacgatgccgtacagcggcgctgggtactgctgaccttga**cgaattagcttcaaaagcgctctgaagttcctatactttctagagaataggaacttcggaataggaacttcaagatcccctgattccctttgtcaacagcaatggatcgaattggccgcggcgttgtgacaatttaccgaacaactccgcggccgggaagccgatctcggcttgaacgaattgttaggtggcggtacttgggtcgatatcaaagtgcatcacttcttcccgtatgcccaactttgtatagagagccactgcgggatcgtcaccgtaatctgcttgcacgtagatcacataagcaccaagcgcgttggcctcatgcttgaggagattgatgagcgcggtggcaatgccctgcctccggtgctcgccggagactgcgagatcatagatatagatctcactacgcggctgctcaaacttgggcagaacgtaagccgcgagagcgccaacaaccgcttcttggtcgaaggcagcaagcgcgatgaatgtcttactacggagcaagttcccgaggtaatcggagtccggctgatgttgggagtaggtggctacgtctccgaactcacgaccgaaaagatcaagagcagcccgcatggatttgacttggtcagggccgagcctacatgtgcgaatgatgcccatacttgagccacctaactttgttttagggcgactgccctgctgcgtaacatcgttgctgctgcgtaacatcgttgctgctccataacatcaaacatcgacccacggcgtaacgcgcttgctgcttggatgcccgaggcatagactgtacaaaaaaacagtcataacaagccatgaaaaccgccactgcgccgttaccaccgctgcgttcggtcaaggttctggaccagttgcgtgagcgcatacgctacttgcattacagtttacgaaccgaacaggcttatgtcaattcgatctagaattattccattgagtaagtttttaagcacatcagcttcaaaagcgctctgaagttcctatactttctagagaataggaacttcggaataggtacttcaagatccccaattcg**accttcttccgtcagatgctggccgatgtcgatgaaccgacggcgccgttcggcttgctggaagtgcatggcgacggcggcggcctggaagaaggccatgtgcgcctcagcgcgaccttgtctcgacgcttgcgccagcaggcgcggcagctgggcgtcagtgcagccagcctgtgccacctggcgtgggcgcaagtgctggcgcgggtcgccaaccgcagcgaggtggtgttcggcaccgtgctgttcggccgtatgcaaggcggcgaaggcgccgaccgcatgatgggcttgctggtcaatacgctgccgctgcggctcaacatcgatacccagggagcggcagccagcgtgcggcatacgcacgccttgctggcgcagctgatggaacacgagcatgcctcgctggcgctggcccagcgcgccagtgcgattgccgcgccgcagcccttgttctcggccttgctcaattatcgtcacagcgtattgggcgaaccctccccggccgagcaggcgatctggcagggcatcacccagatctcgggcgaagagcgcagcaactaccccttgagcctgtcgatcgacgacctcggatcggattttgcgctgaccgcgcaggttacgcccaccgtcggcgcacagcgcgtgtgcggtttcatggcggccgctctggaagggttggtgacagcgctggaggcggagccggaacgcgccgtcaatagcatcgacgtcatgccggcagaagagcggcatcaggtggtcagcaaatAAGCTTTCAAGCAAGCAAAAAAAA

1. Tripropeptin A KO sequence

AAAAAAAATCAAGCAAGCAAGCTTggtcaagatacgcggtttccgtattgaactcggtgagatcgaagcgagattgtcgcgcatcgaaggtatacgcgaaacagtggtgattgcacgggaagacagtccaggcgacaagcgcctggtggcctacatggtggctgagccgggtgcattgcctcctgatccggccgagctgcacgagcaactcaaggcgcaactacctgaatacatggtcccggcagcgtacgtgatactgggatccttgccgctgacacccaatggtaaactcgatcgcaaggcgctgccggcgccggaaggaggcgtcttcatccagcgcgcctacgaggcgccgcagggcgagattgaacaggtgctggcgcagatctggtcggcactgctcggcgtcgaacgtatcggccgtcgcgatcatttcttcgaactgggtggacattcgttgctggccatccggcttgtcgagcaactgcgccgacgcgaatggttcatcgatatccgttccttgttcgcccagcctcaattgtcatccttggcgacagccatccaacagaccgccagcctgggcaaacgcgacgtcgtgccgcctgccaatggcatcccgcaagacgccgcggccatcacgccagccatgctgccgctggccgcattgaatgaaatgcatatcgcacggattgtgcaggcgacgccgggcggcgtcgccaatatccaggacatctatccgctggcgccgctgcaggaaggcatcctgtttcaccatctgctgcaaaccgagggcgacgcctatgtcctgccgaccttgctgggtttcgacagcaaggaccggctcgatcgttttacggccgcactcaacacggttatctcacgccatg**cgaattagcttcaaaagcgctctgaagttcctatactttctagagaataggaacttcggaataggaacttcaagatcccctgattccctttgtcaacagcaatggatcgaattggccgcggcgttgtgacaatttaccgaacaactccgcggccgggaagccgatctcggcttgaacgaattgttaggtggcggtacttgggtcgatatcaaagtgcatcacttcttcccgtatgcccaactttgtatagagagccactgcgggatcgtcaccgtaatctgcttgcacgtagatcacataagcaccaagcgcgttggcctcatgcttgaggagattgatgagcgcggtggcaatgccctgcctccggtgctcgccggagactgcgagatcatagatatagatctcactacgcggctgctcaaacttgggcagaacgtaagccgcgagagcgccaacaaccgcttcttggtcgaaggcagcaagcgcgatgaatgtcttactacggagcaagttcccgaggtaatcggagtccggctgatgttgggagtaggtggctacgtctccgaactcacgaccgaaaagatcaagagcagcccgcatggatttgacttggtcagggccgagcctacatgtgcgaatgatgcccatacttgagccacctaactttgttttagggcgactgccctgctgcgtaacatcgttgctgctgcgtaacatcgttgctgctccataacatcaaacatcgacccacggcgtaacgcgcttgctgcttggatgcccgaggcatagactgtacaaaaaaacagtcataacaagccatgaaaaccgccactgcgccgttaccaccgctgcgttcggtcaaggttctggaccagttgcgtgagcgcatacgctacttgcattacagtttacgaaccgaacaggcttatgtcaattcgatctagaattattccattgagtaagtttttaagcacatcagcttcaaaagcgctctgaagttcctatactttctagagaataggaacttcggaataggtacttcaagatccccaattcg**gcgttaatacagcagggacggcaggccgagcttcctacgcctgtgccgttccgcaattttgtggcacaagcaaggctgggcgtgagcgaagccgagcacgaggatttcttccggcagatgctggcggatgtcgatgaaccgacagcgccgttcggtttgctggatgtgcagggggacggttcccagattgcacaggccagactgatcctgcctttcgagctggcgctgcggctgcggcggcaggctaaaacgcggggattcagcgcggccagcctgttccacctggcctgggcgcaagtgctggcccaatgcacgggccgcgacgacgtggtgttcggcacggtgctgttcggccgcatgcagagtggcgcgggcgcggatcgcgccatcggcttgttcatcaataccttgccgttgcgcgtgaagttaggtgagtgcggcgtcgaggaaggtttgcagcaggtgcatgcggcgttgactgggttgctgcaccacgaacatgcttcactggcgctggcccagcgctgcagtggattgccggtcaatacgccgctattctcggccttgctgaactatcgccatagccacgttgccagcgccgatgaaacggaaattctcgaaggcgtccgcttcctcggggtccgcgaccgcaccaattatcccttcggcttgtacatcgacgattccggccgcgactttgaactgacggtccaggtcgacgagtctgtgtcagcgcagcatatcgccctgtacatgcaacagacgctggaacaattggcattAAGCTTTCAAGCAAGCAAAAAAAA
